# A cocktail of human monoclonal antibodies broadly neutralizes North American rabies virus variants as a promising candidate for rabies post-exposure prophylaxis

**DOI:** 10.1038/s41598-022-13527-0

**Published:** 2022-06-07

**Authors:** Monir Ejemel, Todd G. Smith, Lauren Greenberg, William C. Carson, David Lowe, Yong Yang, Felix R. Jackson, Clint N. Morgan, Brock E. Martin, Chantal Kling, Christina L. Hutson, Nadia Gallardo-Romero, James A. Ellison, Susan Moore, Adam Buzby, John Sullivan-Bolyai, Mark Klempner, Yang Wang

**Affiliations:** 1 MassBiologics of the University of Massachusetts Chan Medical School, 460 Walk Hill, Boston, MA 02126 USA; 2 Poxvirus and Rabies Branch, Division of High-Consequence Pathogens and Pathology, Centers for Disease Control and Prevention, 1600 Clifton Road NE, Atlanta, GA 30329 USA; 3 Veterinary Diagnostic Laboratory, Rabies Laboratory, Kansas State University, Manhattan, KS 66502 USA; 4 Present Address: Veterinary Medical Diagnostic Laboratory, University of Missouri, Columbia, MO 65211 USA; 5 Present Address: Apellis Pharmaceuticals, Waltham, MA USA

**Keywords:** Drug discovery, Immunology, Medical research, Neurology

## Abstract

Human rabies remains a globally significant public health problem. Replacement of polyclonal anti-rabies immunoglobulin (RIG), a passive component of rabies post-exposure prophylaxis (PEP), with a monoclonal antibody (MAb), would eliminate the cost and availability constraints associated with RIG. Our team has developed and licensed a human monoclonal antibody RAB1 (Rabishield^©^), as the replacement for RIG where canine rabies is enzootic. However, for the highly diverse rabies viruses of North America, a cocktail containing two or more MAbs targeting different antigenic sites of the rabies glycoprotein should be included to ensure neutralization of all variants of the virus. In this study, two MAb cocktails, R172 (RAB1-RAB2) and R173 (RAB1-CR57), were identified and evaluated against a broad range of rabies variants from North America. R173 was found to be the most potent cocktail, as it neutralized all the tested North American RABV isolates and demonstrated broad coverage of isolates from both terrestrial and bat species. R173 could be a promising candidate as an alternative or replacement for RIG PEP in North America.

## Introduction

Rabies virus (RABV) is a zoonotic pathogen with both wild and domesticated animal reservoirs. Human infection with RABV leads to acute fatal illness with rapidly progressing encephalitis^1^. Approximately 61,000 people, mostly children, die worldwide from rabies each year, making human rabies a significant public health concern^2^. In Asia and Africa, dog bites account for nearly all human rabies infections^3^. However, in the United States, most human rabies cases are caused by exposure to insectivorous bats and terrestrial animals including skunks and raccoons^4,5^.

The mortality of rabies can be prevented by prompt administration of post-exposure prophylaxis (PEP) consisting of wound care and combined passive and active immunization with rabies immune globulin (RIG) and the rabies vaccine respectively. With proper administration, PEP is effective at preventing disease; and human rabies treatment after the administration of PEP usually deviates from treatment procedures in the setting of severe exposures.

An estimated 36,000 courses of rabies PEP are administered each year in the US^6^. The cost of rabies PEP varies greatly in the US and around the world, with the majority of the cost coming from RIG^7^. RIG is produced from the serum of RABV-immunized human donors (hRIG) or from hyper-immunized horses (eRIG). Worldwide there is a shortage of RIG, due to the expense involved in producing large quantities of a fractionated blood product. Administration of RIG derived from human donors or horses could also pose a potential safety threat due to adventitious agents.

Human monoclonal antibodies (HuMAb) against RABV glycoprotein (G) have been proposed as a replacement for RIG, with several candidates already advanced to clinical stages^8^. Our team has developed and characterized a HuMAb, RAB1, licensed as Rabishield©, as a RIG replacement for PEP in India where canine rabies is enzootic^9,10^. An analysis in India demonstrated that the glycoprotein amino acid residues required for human monoclonal antibody RAB1 neutralization are conserved in all RABV isolates in terrestrial animals. This suggests a single monoclonal antibody (MAb) is sufficient for rabies PEP in regions where nearly all rabies infections are caused by terrestrial isolates^9,10^.

The major challenge for the development of a MAb product for rabies PEP in North America is the high diversity of RABV variants in both terrestrial and bat isolates. There are seven RABV variants that circulate regionally in terrestrial mammals in the US: raccoons, north central skunks, south central skunks, Arizona gray foxes, California skunks, arctic foxes, and dogs-mongooses^4^. In Canada and Mexico, the RABV variants circulate in similar terrestrial reservoirs, including skunks in Canada and Mexico, gray foxes in Mexico, and red arctic foxes in Canada^4^. In addition to terrestrial reservoirs, nearly every bat species harbors one or more RABV variants with vampire bat *Desmodus rotundus* being the variant of concern in Mexico^11^. Recent human rabies cases in the US were predominately caused by three bat RABV variants: silver-haired bat *Lasionycteris noctivagans* (Ln), tri-colored bat *Perimyotis subflavus* (Ps), and Mexican free-tailed bat *Tadarida brasiliensis* (Tb)^4^.

RIG is generally available in the United States. However, to prevent potential RIG shortages and eliminate the theoretical risk of blood-borne transmission of serum products, MAbs are being developed as an alternative to RIG for rabies PEP. Based on the diversity of RABV bat variants, a cocktail of more than one MAb is likely required to ensure neutralization of all variants in North America^12^. The World Health Organization (WHO) has recommended that if MAbs are being considered for the prevention of rabies, then at a minimum, two MAbs targeting different antigenic sites of the G protein should be included the use of antibody cocktails^13^. A document was also recently published by the FDA as a guide for commercial development of such antibody cocktails^14^. For this reason, the focus of this study was to develop and characterize two MAbs recognizing different and non-competing antigenic sites on the RABV envelope glycoprotein. Here, we developed two anti-rabies MAb cocktails, R172 and R173. Both cocktails were evaluated against a broad range of RABV variants in North America. R173 was found to provide complete coverage against all tested RABV isolates from terrestrial and bat species in North America.

## Materials and methods

### Antibody cloning, expression and purification

RAB1 and RAB2 were previously developed as anti-rabies glycoprotein human monoclonal antibodies isolated from human IgG transgenic mice immunized with rabies vaccine as previously described^9^. The heavy chain and light chain variable regions of RAB1 and RAB2 were amplified from hybridoma cells by RT-PCR and cloned into a pcDNA 3.1 (Life Technologies) vector containing the heavy or light constant regions of human IgG1. For MAb CR57, the variable region sequences of light chain (GenBank: CS239779), and heavy chain (GenBank: CS239777), were synthesized (GenScript Inc) and cloned into IgG1 expression vectors as described above.

Antibodies were expressed in CHO cells stably transfected with DNA encoding RAB1, RAB2, or CR57 IgG1. The cell supernatants were harvested by centrifugation, and then incubated with protein A Sepharose resin (GE Healthcare) for 2 h at room temperature. The beads were washed with phosphate buffered saline (PBS) and the antibody was eluted with 100 mM glycine pH 2.8. The antibody was then dialyzed into PBS storage buffer. Purified antibody was filter sterilized and the protein concentration was determined by spectrophotometry.

### Cell culture

HEK-293 T/17 cells were grown in Dulbecco's Modified Eagle Medium (DMEM) (ThermoFisher scientific) supplemented with 10% Fetal bovine serum (FBS) (Millipore Sigma) and 100 IU of penicillin–streptomycin (ThermoFisher scientific) at 37ºC with 5% CO_2_. The HEK293T/17 cells were harvested using PBS containing 5 mM EDTA (UltraPure™, pH 8.0, ThermoFisher) and then incubated for 5 min at room temperature. Human osteosarcoma (HOS) cells were grown in minimum essential medium (MEM) (ThermoFisher) supplemented with 10% FBS, 2% L-Glutamine, 2% MEM Vitamins, 1% penicillin–streptomycin (Gibco), and 1% sodium bicarbonate and then incubated at 37 °C with 5% CO_2_. Cells were harvested using trypsin–EDTA (0.25%) (ThermoFisher).

### Generation of RAB2 resistant viruses

The method to generate RABV resistant viruses was described in our previous study^9^. Briefly, mouse neuroblastoma cells were plated at 1.5 × 10^5^ cells/ml on Day 1. On Day 2, a titration of 1 × 10^1^ to 10^8^ FFU/ml/well of CVS-11 RABV was incubated with 50 ug/ml of RAB2 at 37 °C for 1 h. The virus/RAB2 mix was added to cells and incubated at 37 °C. 12 h later, cell media was exchanged with fresh media containing 50ug/mL RAB2. Cells were incubated for additional 3-days at 37 °C. On Day 5, supernatant containing potentially resistant virus was harvested and stored at 4 °C. The cells were fixed and stained for RFFIT. Virus taken from wells containing 1–5 fluorescent foci were further amplified on MNA cells for 3 days in the presence of RAB2. RNA was extracted from virus-infected cells for RT-PCR and sequencing analysis for the potential mutations in glycoprotein-encoding genes. The sequencing result was confirmed by two separate infections/RNA preparations to confirm the observation.

### Cloning of rabies virus ERA glycoproteins

The gene sequence encoding glycoproteins of RABV Evelyn-Rokitnicki-Abelseth (ERA) was codon-optimized for mammalian expression as described previously^9,15^. The amino acid (a.a.) sequence of the RABV glycoprotein full length surface protein (a.a. 1–524), as well as its soluble fragment (a.a 1–439), were cloned into an expression vector pcDNA3.1Myc/His (ThermoFisher). Proteins were expressed in frame with the myc/his tags. Alanine scanning mutants were generated using site-directed mutagenesis. Overlapping primers containing the desired point mutations were used to amplify full-length mutant glycoprotein genes and the pcDNA3.1Myc/His vector from the previously cloned codon-optimized ERA RABV glycoprotein. PCR positive clones were selected according to the manufacturer’s recommendation (New England BioLabs® Site-Directed Mutagenesis Kit Protocol (E0554)).

### Recombinant glycoprotein expression and purification

All constructs were transfected into HEK-293 /17(ATCC) cells using a 1:4 DNA-Polyethylenimine (PEI) (Sigma) ratio, as described by the manufacturer. Cells were grown to 95% confluence in 150 mm tissue culture dishes in 30 mL of DMEM-10% (FBS) (Sigma). 30 µg of DNA mixed with 120 µl of PEI was added to the cells, and the plates were incubated overnight at 37 °C. The media was removed and stored at 24-, 48- and 72-h post-transfection for secreted soluble proteins or was discarded for membrane bound proteins.

Soluble rabies glycoproteins containing Myc and His epitope tags were purified from the cell culture supernatant by incubation with nickel-nitrilotriacetic acid (Ni–NTA) beads (ThermoFisher) for 2 h, followed by column filtration using 10 mM imidazole to washg non-specific binding off of the column, and 250 mM imidazole for the final elution of the protein. The eluted proteins were then dialyzed for two hours in PBS at pH 7.

### Affinity determination for RAB2

Bio-layer interferometry (BLI) with an Octet HTX (PALL/ForteBio) was used to determine the affinity of RAB2 IgG for the soluble glycoprotein of ERA. RAB2 was added to 96 well plates at 1000 nM and titrated 1:2 to 62 nM in PBS. Purified RAB2 antibody was immobilized on anti-human IgG Fc Capture (AHC) Biosensors (ForteBio) for 120 s at 333 nM. After a baseline step, the RAB2-antigen binding rate was determined when the biosensors with immobilized antibody were exposed to ERA glycoprotein at different concentrations for 120 s. Following the association step, the soluble RAB2-ERA glycoprotein complex was exposed to PBS and the rate of the ERA glycoprotein dissociation from RAB2 was measured. Each assay was performed in triplicate. The binding affinity of RAB2 for the ERA glycoprotein was calculated using association and dissociation rates with ForteBio Data analysis software v8.1 (PALL).

### Cell surface staining

HEK-293T/17 cells were transfected with constructs encoding the full-length wild type glycoprotein of ERA or with individual alanine mutants. Cells were harvested 48 h post-transfection and incubated with varying concentrations of HuMabs. The binding activities of HuMAb to glycoproteins was detected by phycoerythrin labeled anti-human IgG (Jackson) in flow cytometry (MACSQuant), and analyzed with MACSQuant® Analyzer 10 (Miltenyi Biotec®).

### ELISAs

ELISA plates were coated with ERA soluble glycoproteins (a.a. 1–439). Plates were washed and then blocked with ELISA blocking buffer (EBB; PBS, 1% BSA, 0.05% Tween). Antibody samples were added to the plate and then diluted 1:2 in EBB. Samples were detected with a goat anti-human IgG conjugated with Alkaline Phosphatase (1:500, Jackson ImmunoResearch). The plates were developed with p-Nitrophenyl phosphate disodium salt at 1 mg/mL in 1 M diethanolamine (ThermoFisher) for 20 min, and then analyzed at 405 nm with a Vmax plate reader (Molecular Devices).

### Production of pseudotyped viruses and neutralization assay

A plasmid containing a replication defective lentivirus backbone (Env-, Vpr- HIV; HIV resources) fused with the firefly luciferase gene, pNL4-3.Luc.R-E-, was co-transfected with a plasmid encoding RABV glycoprotein ERA into 293 T cells using PEI as described previously^16,17^. Following a media change 24 h post-transfection, pseudoviral particles were harvested 48–72 h post-transfection, concentrated, and stored at − 80 °C. An infection assay was performed to determine the luciferase counts per second (cps) of the pseudovirus preparations. 24 h post-infection, the media was changed to DMEM without phenol red (ThermoFisher) with 10% FBS and 1% Penicillin–Streptomycin. Luciferase activity was detected 72 h post-infection using the Bright-Glo Luciferase Assay System (Promega) and analyzed by Victor3 de nivo multilabel plate reader (PerkinElmer). Neutralization assays were performed with approximately 50,000 cps of pseudovirus and incubated for 1 h at room temperature with a 1:5 serial dilution of antibody mix starting at 37 ng/ml. The antibody/virus suspension was then applied to HOS cells (ATCC# CRL-1543) which were then incubated at 37ºC with 5% CO_2_. Luciferase activity was assayed 72 h post-infection using the Bright-Glo reagent (Promega), according to the manufacturer’s protocol. Neutralization results are expressed as the percent inhibition of the baseline luciferase activity.

### In vitro RABV RFFIT neutralization

The rapid fluorescent focus inhibition test (RFFIT) was performed as described previously^18^. MAbs were diluted to a standard starting concentration then serially diluted fivefold. The starting concentration of MAb was increased until neutralization was detected within the dilution range. Different RABV isolates or laboratory strains (including CVS-11) were added to the MAb dilutions. The starting concentration of each virus was empirically determined to result in 50 × 50% fluorescent foci doses (50 FFD_50_) with an acceptable range of 32–100 FFD_50_. For each virus, standard rabies immune globulin (SRIG, US FDA lot R-3) at 2 IU/mL, HyperRab (HRIG, Grifols, Barcelona, Spain) at > 150 IU/mL, each individual MAb, and a 1:1 mixture (cocktail) of the component MAb was tested.

### In vivo efficacy in the Syrian hamster model

Experimental protocols were approved by the CDC’s Institutional Animal Care and Use Committee (#2622SMIHAMC-A18) prior to starting and strictly followed to ensure animal welfare. All methods were carried out in accordance with ARRIVE guidelines and regulations (https://arriveguidelines.org). Four-week-old female Syrian hamsters were purchased from Charles River Laboratory (Wilmington, MA, USA) or Envigo (Indianapolis, IN, USA) and arbitrarily assigned to groups containing 12 or 21 animals depending on the RABV variant used. Animals underwent a three-day quarantine/acclimation period and veterinary examination prior to starting. On day 0, animals were anesthetized with isoflurane (3–5%), and labeled with subcutaneous injection of a preprogrammed passive integrated transponder. While still under anesthesia, the animals were weighed, 0.15 mL of blood was collected using the subclavicle method, and 0.1 mL of RABV was injected intramuscularly (IM) in the gluteal region of the left hind leg. Virus titration data was previously determined in hamsters with various isolates. Each dose is titrated to cause > 80% mortality in untreated and vaccine alone groups. Challenge doses and groups sizes were as follows: canine RABV variant, TX coyote 323R, 10^3.5^ MICLD_50_, n = 12; fox RABV isolate, AZ fox 2400L, 8.6 × 10^2^ ffu, n = 21; Bat RABV variant, PA bat EF A06-3684, 1.4 × 10^3^ ffu, n = 21. Animals were monitored every five minutes until they fully recovered from anesthesia and then once daily until 7 days post-infection (pi).

PEP was initiated 24 h pi and consisted of 20 IU/kg of HyperRab (HRIG, Grifols, and Barcelona, Spain) as a positive control. 1.1 mg/kg of an irrelevant HuMAb was used as a negative control. 1.1 mg/kg of HuMAb cocktail R172, 0.1 mg/kg of R172, or 0.01 mg/kg of R172 was injected IM in the same leg as the challenge virus.0.05 mL of the Imovax rabies vaccine (Sanofi Pasteur, Lyon, France) was injected IM in the opposite leg. One group did not receive any PEP. Rabies vaccine administration was repeated for the surviving animals 4, 8, 15, and 29 days pi (days 3, 7, 14, and 28 of PEP). Blood collection under anesthesia was repeated on days four and eight pi (days 3, and 7 of PEP). The PEP injection site was assessed daily until day 8 pi for any localized reactions.

From days 7 to 21 pi, animals were observed twice daily and were euthanized by isoflurane overdose at the first clinical signs of rabies, according to established euthanasia criteria, or as deemed necessary by the attending veterinarian. From day 22 pi until the experimental endpoint, animals were observed once daily. All surviving animals were euthanized 45 days pi.

All animals that were euthanized with clinical signs and approximately half of the animals surviving to day 45 were tested for RABV antigen in the brain stem by a standard direct fluorescent antibody (DFA) test^19^. For selected animals, RNA was extracted from the brain stem and tested for RABV RNA using the LN34 assay as described previously^20^ or converted to cDNA for RABV sequencing. Serum was separated from the collected blood and used to measure virus neutralizing antibodies in a standard RFFIT^18^.

### Statistical analysis

All statistical calculations were performed using GraphPad Prism version 8.1.1 (GraphPad Software, La Jolla, CA). EC_50_ and IC_50_ values were calculated by sigmoidal curve fitting using nonlinear regression analysis. Survival analysis was calculated from a Kaplan–Meier curve using a Mantel-Cox test. *p* < 0.05 was considered significant.

### Data availability and method declaration

The datasets generated and/or analyzed during the current study are available from the corresponding author upon reasonable request. All methods were performed in accordance with the relevant guidelines and regulations.

## Results

### RAB1 epitope analysis among rabies virus isolates in North America

Our previously work identified a human monoclonal antibody, HuMab RAB1 that recognize a conformational epitope on the RABV glycoprotein, which includes antigenic site III^10^. Residues 336 and 346 were found to be the most critical residues for RAB1 binding to the glycoprotein^9,10^. To further understand the epitope coverage of RAB1 against RABV isolates from North America, including Canada and Mexico, a total of 553 glycoprotein sequences of RABV isolates were analyzed with a focus on the two epitope residues a.a. 336 and 346 (Tables 1, S2). Overall, the region encompassing amino acids 336–346 is highly conserved across all isolates. The variability found in residues 336 and 346 was shown in Table 1 along with their RAB1 neutralization activities in the rapid fluorescent focus inhibition test (RFFIT). Among the terrestrial carnivore isolates in North America, RAB1 epitope residue 336 was asparagine (N) in 89% of total isolates and serine (S) in 11% of total isolates. N336 was paired with arginine (R, 67%) or lysine (K, 22%), and S336 was paired with arginine (R, 11%) at position 346. Isolates with N336 R346, S336 R346 and N336 K346 were all neutralized by RAB1 in RFFITs with EC_50_ values of less than 1 ug/ml (Table 1).

**Table 1:** RAB1 epitope analysis of North America rabies virus isolates.

336	346	Epitope frequency in GenBank (%)^a^	RFFIT neutralization by RAB1
**North American terrestrial rabies virus isolates**			
Asparagine (N)	Lysine (K)	22%	++
Asparagine (N)	Arginine (R)	67%	++
Serine (S)	Arginine (R)	11%	++
**North American bat rabies virus isolates**			
Asparagine (N)	Lysine (K)	11%	++
Asparagine (N)	Arginine (R)	74%	++
Aspartic acid (D)	Arginine (R)	5%	++
Asparagine (N)	Serine (S)	3%	**+**
Asparagine (N)	Glutamic acid (E)	7%	−

A total of 553 sequences was analyzed for North America RABVs including both terrestrial and bat isolates.

++EC_50_ < 1 µg/mL.

**+**EC_50_ > 1 µg /mL.

− Not neutralized by HuMAb at the highest concentration tested.

^a^The occurrence frequency of RAB1 epitope with the noted residues at positions 336 and 346. Isolates with noted epitope variants were tested in RFFIT.

Among the North American bat RABV isolates, N336 can be paired with arginine (R, 74%), lysine (K, 11%), glutamic acid (E, 7%) or serine (S, 3%), and D336 can be paired with Arginine (R, 5%) at position 346.The RAB1 antibody was shown to neutralize most N336 E346 isolates but not isolates from arboreal bats (Table 1). Taken together, our analysis suggests RAB1 alone is insufficient to cover the epitope diversity of RABV isolates in North America. Therefore, an antibody cocktail is needed.

### Selection and characterization of an additional HuMAb, RAB2 as a cocktail component

**Figure 1:**
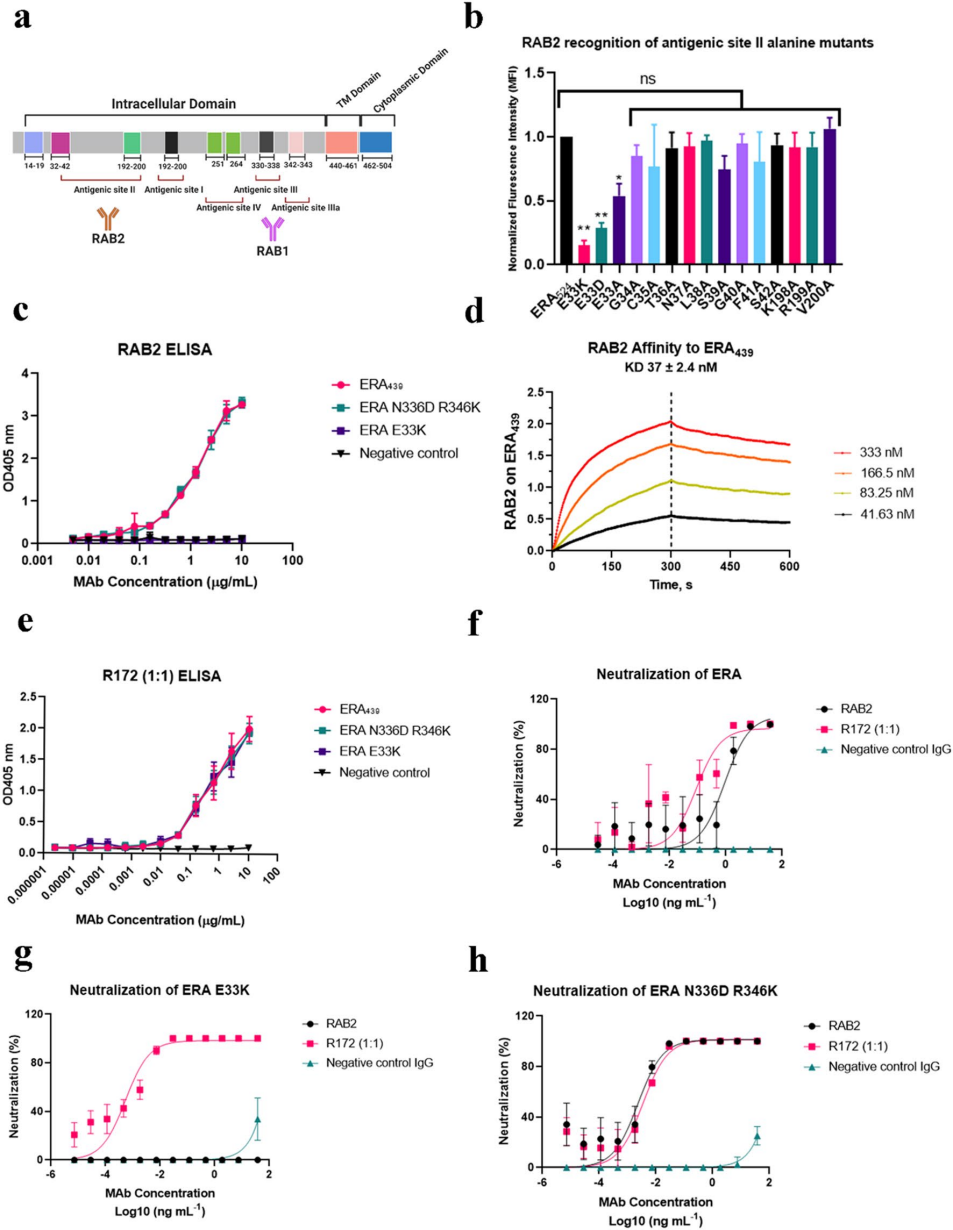
RAB2 and R172 binding and neutralization of rabies glycoproteins. (**a**) Schematic of rabies glycoprotein showing non-overlapping antigenic site locations of RAB1 (antigenic site III) and RAB2 (antigenic site II), figure was created with BioRender.com. (**b**) RAB2 recognition of antigenic site II alanine mutants. Each amino acid in the antigenic site II of ERA full length surface glycoprotein (a.a. 1–524) was substituted with alanine by site directed mutagenesis. (**c**) ELISA binding of RAB2 to the wild-type ERA439 or RAB1 escape N336D R346K and RAB2 escape E33K mutants. (**d**) Affinity measurement of RAB2 antibody against wild type ERA439, conducted by Bio-layer interferometry. (**e**) ELISA binding of R172 to the wild-type ERA439 or RAB1 escape N336D R346K and RAB2 escape E33K mutants. (**f**–**h**) R172 pseudovirus neutralization against lentivirus bearing the wild-type ERA glycoprotein (**f**), E33K (**g**) and N336D R346K (**h**) mutants. ERA glycoprotein was used as backbone to introduce all mutants. EC50 values were calculated by nonlinear regression analysis using Prism version 8.1.1. Data is plotted as the mean ± s.d. from n = 4 independent experiments.

To identify an additional MAb for the cocktail, we started with the HuMAb panel from which RAB1 was previously chosen. We selected RAB2, the 2nd most potent HuMAb from the panel^12^. To understand the epitope of RAB2, resistant virus was generated using CVS-11 strain in the presence of 50 ug/ml of RAB2. Sequencing of the RAB2 escape virus revealed a glutamic acid to lysine change at position 33 (E33) within the antigenic site II. To confirm this finding and assess additional residues for the RAB2 epitope, a series of alanine residues were introduced in the glycoprotein in antigenic site II—including positions 33–42 and 198–200 of the RABV ERA strain (Fig. 1a). Glycoproteins from alanine scanning mutagenesis were expressed on the surface of HEK-293 T/17 cells, stained with fluorescently labeled RAB1 and RAB2, and analyzed by flow cytometry. RAB1 binding was used to normalize protein expression to look at RAB2 binding against the escape mutants. Position 33 was confirmed to be critical for RAB2 binding, but none of the other adjacent residues were shown to be important for RAB2 binding (Fig. 1b). The RAB2 binding to ERA E33K (*p* = 0.001) and E33D (*p* = 0.0017) was disrupted as confirmed by ELISA and flow cytometry (Fig. 1c). The KD affinity value of RAB2 against the ERA glycoprotein was determined to be 37 nM ± 2.4 nM by biolayer interferometry using anti-human Fc capture tips (Fig. 1d). By comparison, the affinity of RAB1 against the ERA glycoprotein was previously determined to be KD = 2.82 nM ± 0.5 nM^9^.

### R172 binding and neutralization of escape mutants

The above epitope analysis suggests that RAB2 binds with high affinity to a different antigenic site than RAB1. A formulated RAB1 and RAB2 cocktail (1:1 mixing ratio, named R172) was then tested against the RAB2 escape mutant E33K in ELISA binding assays. In addition, a previously generated laboratory-derived escape mutant of RAB1, N336D R346K, was included in the assays to demonstrate the cross coverage of RAB2^10^. The R172 cocktail was found to bind the RAB1 escape (N336D R346K) and RAB2 escape mutants (E33K) (Fig. 1e) on a similar level as the wild type (ERA_439_). The EC_50_ value of R172 ELISA binding to the wild type was 0.41 µg/mL, E33K was 0.35 µg/mL, and N336D R346K was 0.34 µg/mL (Fig. 1f).

To confirm binding results, a neutralization assay was performed testing RAB2 against RABVpp pseudovirus containing E33K (Fig. 1g) or N336D R346K (Fig. 1h). The RABVpp was generated from a replication-defective HIV lentivirus backbone engineered to contain the RABV ERA glycoprotein. An irrelevant IgG1 HuMAb was included as the negative control. RAB2 potently neutralized ERA wild type ERA (EC_50_ = 0.76 ng/ml), ERA N336D R346K (EC_50_ = 2 pg/ml) but not ERA E33K. By contrast, the R172 cocktail had a potent neutralizing activity against the wild type ERA (EC_50_ = 0.22 ng/ml), N336D R346K (EC_50_ = 3 pg/ml) and E33K (EC_50_ = 0.5 pg/ml).

### In vitro RABV neutralization of R172 by RFFIT

R172 was further tested by RFFIT against 30 RABV authentic variants isolated in North America, including terrestrial and bat isolates. Viruses were grouped based on the predicted sequence of the MAb epitopes (Tables 2 and 3). For terrestrial isolates, three epitope variants were identified including E33 N336 R346 (ENR), E33 N336 K346 (ENK), E33 S336 R346 (ESR) and E33 D336 R346 (EDR). R172 neutralized all terrestrial isolates with an average EC_50_ in the low ng/mL range (Tables 2, S1). For bat isolates, more epitope variants were identified including E33 N336 R346 (ENR), E33 N336 K346 (ENK), E33 N336 S346 (ENS), E33 D336 R346 (EDR) and 33D 336 N 346E (DNE). R172 was able to neutralize all bat strains in low ng/ml range EC_50s_ except for the 33D 336 N 346E (DNE) isolates.

**Table 2:** RFFIT of RAB1, RAB2 and R172 against North America terrestrial isolates.

Viral isolate	Source (location)	Epitope^b^	RAB1	RAB2	R172	HRIG
TX Coyote 323R	Coyote (Texas)	ENR	++	**+**	++	54
AK Fox	Fox (Alaska)	ENR	++	++	++	78
CA SK	Skunk (California)	ENR	++	++	++	48
PR Mong	Mongoose (Puerto Rico)	ENR	++	++	++	86
Sonora Dog	Dog (Mexico)	ENR	++	++	++	50
TXSK 4380	Skunk (Texas)	ENR	++	++	++	175
TXSK 5171	Skunk (Texas)	ENR	++wt	++	++	26
			−^a^			
ERA	Lab Strain	ENR	++	++	++	67
NCSK	Skunk (North-central US)	ENK	++	++	++	49
TXFX	Fox (Texas)	ENK	++	++	++	40
RAC	Raccoon (Southeast US)	ESR	++	++	++	37
CVS-11	Lab Strain	EDR	**+**	++	++	25

++EC_50_ < 1 µg/mL.

**+**EC_50_ ≥ 1 µg /mL.

− Not neutralized by HuMAb at the highest concentration tested.

HRIG potency IU/mL against given isolate.

^a^Lab-acquired mutation I338T identified in TKSK 5171 following additional cell culture passage. A primary wild type (wt) isolate tested.

^b^Epitope variants with the noted residues at positions 33, 336, and 346.

**Table 3:** RFFIT of RAB1, RAB2 and R172 against North America bat isolates.

Viral isolate	Source (location)	Epitope^b^	RAB1	RAB2	R172	HRIG
DR Brazil	*Desmodus rotundus* (Brazil)	ENR	++	++	++	34
C1434	*Perimyotis subflavus* (Alabama)	ENK	++	++	++	40
CO/EF Bat 6938	*Eptesicus fuscus/Myotis* sp. (Colorado)	ENK	++	++	++	64
WA Bat	*Lasionycteris noctivagans* (Washington)	ENK	++	++	++	44
AZ Fox 2400	Ef variant in a fox (Arizona)	ENK	++	++	++	139
AZ 3860 Bat^a^	*Parastrellus hesperus* (Arizona)	ENS	−	++	++	38
CA Bat	*Myotis thysanodes* (California)	ENS	++	++	++	10
Myotis	Myotis sp.(Washington)	ENS	++	++	++	26
1625 Bat	*Lasiurus borealis* (Michigan)	DNE	**+**	**+**	**+**	15
AZ Bat LC	*Aeorestes cinereus* (Arizona)	DNE	**+**	**+**/−	**+**	10
FL Bat 769	*Lasiurus seminolus* (Florida)	DNE	**+**	−	**+**/−	6
TN132	*Lasiurus borealis* (Tennessee)	DNE	**+**/−	−	**+**/−	11
TN269	*Lasiurus borealis* (Tennessee)	DNE	**+**/−	−	**+**/−	9
TN33	*Lasiurus borealis* (Tennessee)	DNE	**+**	**+**/−	**+**	9
TN410	*Aeorestes cinereus* (Tennessee)	DNE	**+**	**+**	**+**	11
VA1340	*Lasiurus borealis* (Virginia)	DNE	**+**/−	−	**+**/−	8
VA399	*Lasiurus borealis* (Virginia)	DNE	**+**	**+**/−	**+**	6
Bat EF	*Eptesicus fuscus* (Pennsylvania)	EDR	++	++	++	46

++EC_50_ < 1 µg /mL.

**+**EC_50_ > 1 µg /mL.

**+**/− EC_50_ > 100 ug/ml.

− Not neutralized by HuMAb at the highest concentration tested.

HRIG potency IU/mL against given isolate.

^a^Lab-acquired mutation I338T identified in 3860 Bat following cell culture adaptation. A primary (wild type) isolate of this virus is not available to test.

^b^Epitope variants with the noted residues at positions 33, 336, and 346.

For the DNE isolates, RAB1 neutralized with an average EC_50_ of 44 µg/mL, RAB2 neutralized with an EC_50_ approximately tenfold higher at 500 µg/mL, and R172 neutralized with an average EC_50_ in the 60 µg/mL range (Tables 3, S1). HRIG was used for comparison in the RFFIT assay. Because HRIG is a polyclonal product, comparing the EC_50_ is problematic. Instead, the 50% endpoint titer was used for comparison. The neutralization of viruses in the different epitope classes via HRIG roughly followed R172 neutralization, in that HRIG was more potent against ENS, ENK, ENR, EDR, and ESR viruses than against DNE isolates.

### In vivo R172 efficacy in the Syrian hamster model

To assess the in vivo efficacy of R172, hamsters were challenged with a canine terrestrial variant TX coyote 323R; a fox isolate AZ Fox 2400L, or a bat isolate, PA bat EF A06-3684 (RAB1 and RAB2 epitopes are as listed in Table 3). Standard PEP was administered 24 h later with five doses of rabies vaccine (days 0, 3, 7, 14, 28) as well as different doses of HuMAb cocktail R172, or the standard of care (20 IU/kg HRIG), or 1.1 mg/kg irrelevant HuMAb on day 0. For all the isolates, the survival curve for the group that received irrelevant HuMAb and vaccine was not significantly different from the control group that received no treatment (*p* = 0.92, 0.68, 0.12, and 0.15 respectively; Fig. S1).

For the canine RABV variant (TX coyote 323R), survival curves for the 1.1, 0.1, and 0.01 mg/kg R172 groups were not significantly different from the standard of care HRIG group (*p* = 0.07, 0.92, 0.16 respectively). For the fox isolate (AZ Fox 2400L), survival curves for the 1.1 and 0.1 mg/kg R172 groups were significantly better than the standard of care HRIG group (*p* = 0.01, 0.014 respectively), and the 0.01 mg/kg R172 group was not significantly different from the standard of care (*p* = 0.064) but was significantly better than the irrelevant HuMAb group (*p* < 0.0001). For the bat isolate (PA bat EF A06-3684), the survival curve for the 1.1 mg/kg R172 group was significantly better than the standard of care HRIG group (*p* = 0.001). The survival curve for the 0.1 mg/kg R172 group was not significantly different from the standard of care HRIG group (*p* = 0.1) but was significantly better than the irrelevant HuMAb group (*p* < 0.0001). The survival curve for the 0.01 mg/kg R172 group was significantly worse than the standard of care HRIG group (*p* = 0.038).

To test R172 against difficult to neutralize RABV isolates with the DNE epitope, all DNE isolates listed in Table 3 were tested in 13 challenge studies. None of the DNE isolates was able to cause mortality in Syrian hamsters. Therefore, DNE isolates could not be used to evaluate the in vivo activity of R172.

### In vitro CR57 and R173, binding and pseudovirus neutralization of RAB1 escape mutants

**Figure 2:**
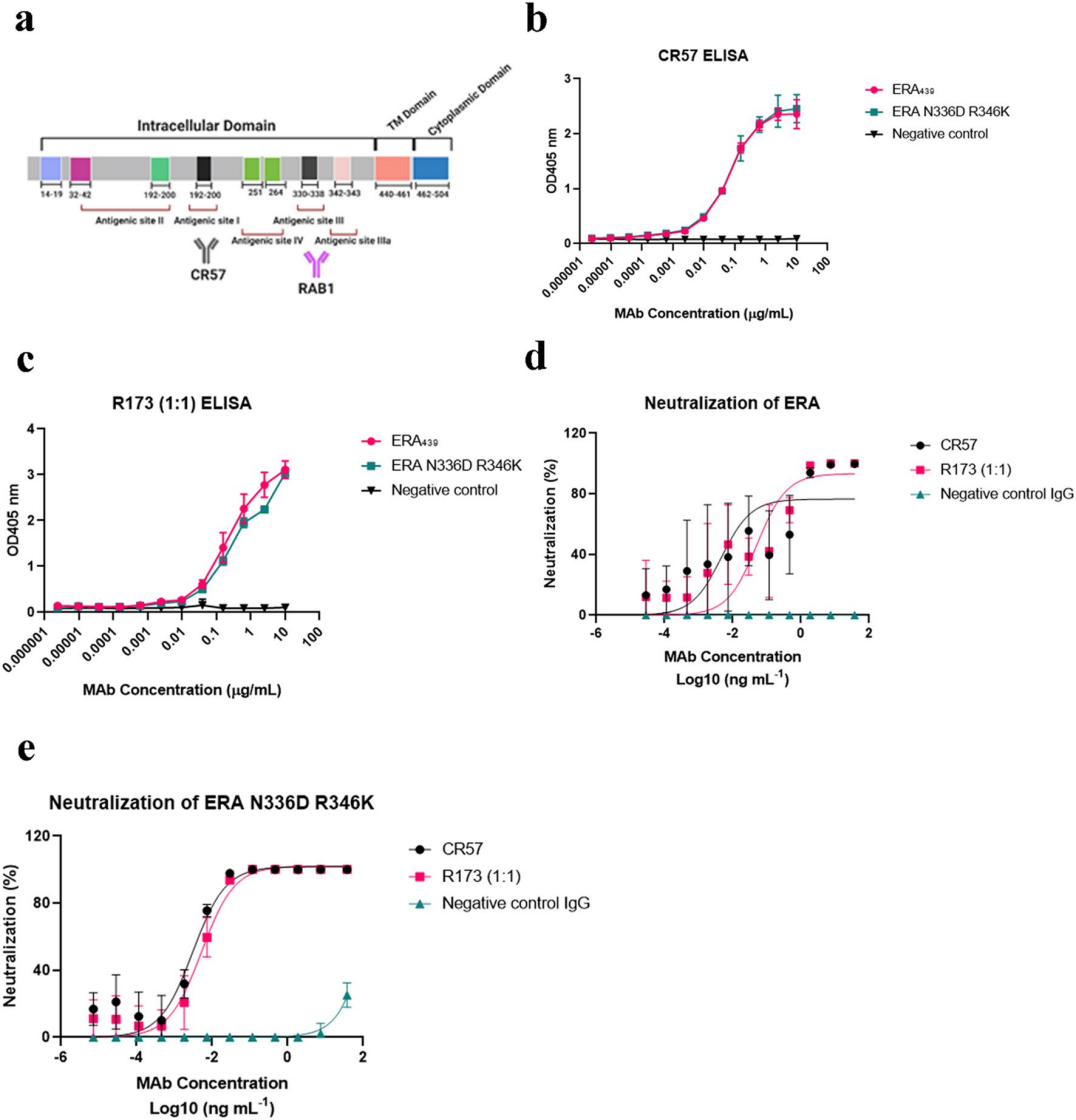
CR57 and R173 binding and neutralization of rabies glycoproteins. (**a**) Schematic of rabies glycoprotein showing non-overlapping antigenic sites locations of RAB1 (antigenic site III) and CR57 (antigenic site I), figure was created with BioRender.com. (**b**, **c**) ELISA binding of CR57 (**b**) and R173 (**c**) to the wild type ERA_439_ or RAB1 escape mutant N336D R346K. (**d**, **e**) Pseudovirus neutralization of RAB1, CR57, and R173 against lentivirus bearing the wild type ERA glycoprotein (**d**) and N336 R346K mutant (**e**). EC_50_ values were calculated by nonlinear regression analysis using Prism version 8.1.1. Data is plotted as the mean ± s.d. from n = 4 independent experiments.

Given the apparently poor neutralization of R172 against bat isolates with the DNE epitope, and the inability to evaluate protection from these variants in vivo, we explored the possibility of using a previously described HuMAb (CR57) to replace RAB2 in the cocktail. CR57 is a human monoclonal antibody with good in vitro and in vivo neutralizing potency against a broad spectrum of RABV variants, as previously described^21^. The CR57 antibody epitope binds to antigenic site I of the RABV glycoprotein (Fig. 2a). CR57 and R173 (RAB1 + CR57 at 1:1) were evaluated against RAB1 escape mutants in both ELISA and pseudovirus neutralization assays. CR57 EC_50_ binding was comparable to the wild type ERA_439_ as well as RAB1 escape N336D R346K with an average EC_50_ value of 0.0001 µg/mL (Fig. 2b). The EC_50_ value of R173 binding was 0.002 µg/mL against the wild type ERA_439_ and 0.005 against RAB1 escape mutant N336D R346K (Fig. 2c). Both CR57 and R173 were able to fully neutralize the wild type ERA_439_ and N336D R346K with EC_50_ ranging between 3 and 5 pg/ml (Fig. 2d,e).

### In vitro RABV neutralization of R173 by RFFIT

RAB1, CR57, and R173 were screened against 17 RABV variants or laboratory strains in the RFFIT. CR57 strongly neutralized the virus with the DNE epitope including *Lasiurus borealis*, *Lasiurus seminolus*, and *Aeorestes cinereus* isolates with an average EC_50_ of 0.033, 0.103, and 0.036 µg/mL respectively (Tables 4 and 5, S1). Two isolates not neutralized by CR57 were effectively neutralized by RAB1: AL bat (Tb variant) and TX Skunk 4380 (South central skunk variant with a lab acquired escape mutation). Another South central skunk variant isolate (TX Skunk 5171 with a lab acquired escape mutation) was neutralized by CR57 but not RAB1. Similar to previous results, neutralization of each isolate with the R173 cocktail was equivalent to the most effective HuMAb in the cocktail. Taken together, the R173 cocktail strongly neutralized all RABV isolates tested, including viruses with the previously difficult to neutralize DNE epitope (Tables 3 and 5, S1). The Ln, Ps, and Tb RABV variants are highly relevant to public health since the majority of human rabies cases in the US in the last 30 years can be attributed to these bat variants^5^.

**Table 4:** RFFIT of RAB1, CR57 and R173 against North America terrestrial isolates.

Viral isolate	Source (location)	Epitope^b^	RAB1	CR57	R173	HRIG
TXSK 4380	Skunk (Texas)	NR*	++	++wt	++	x
				−^a^		
TXSK 5171	Skunk (Texas)	NR*	++wt	++	++	x
			−^a^			
CVS-11	Lab Strain	DR	**+**	++	++	x

++EC_50_ < 1 µg/mL.

**+**EC_50_ ≥ 1 µg /mL.

− Not neutralized by HuMAb at the highest concentration tested.

x Neutralized by HRIG.

^a^Lab-acquired mutation following additional cell culture passage. A primary wild type (wt) isolate was tested.

^b^RAB1 epitope variants with the noted residues at positions 336, and 346.

**Table 5:** RFFIT of RAB1, CR57 and R173 against North America bat isolates.

Viral isolate	Source (location)	Epitope^b^	RAB1	CR57	R173	HRIG
C1434	*Perimyotis subflavus* (Alabama)	NK	++	**+**	++	x
CO/EF Bat 6938	*Eptesicus fuscus/Myotis* sp. (Colorado)	NK	++	++	++	x
WA Bat	*Lasionycteris noctivagans* (Washington)	NK	++	++	++	x
AZ 3860 Bat^a^	*Parastrellus hesperus* (Arizona)	NS	−	++	++	x
1625 Bat	*Lasiurus borealis* (Michigan)	NE	**+**	++	++	x
AZ Bat LC	*Aeorestes cinereus* (Arizona)	NE	**+**	++	++	x
FL Bat 769	*Lasiurus seminolus* (Florida)	NE	**+**	++	++	x
TN132	*Lasiurus borealis* (Tennessee)	NE	**+**	++	++	x
TN269	*Lasiurus borealis* (Tennessee)	NE	**+**	++	++	x
TN33	*Lasiurus borealis* (Tennessee)	NE	**+**	++	++	x
TN410	*Aeorestes cinereus* (Tennessee)	NE	**+**	++	++	x
VA1340	*Lasiurus borealis* (Virginia)	NE	**+**	++	++	x
VA399	*Lasiurus borealis* (Virginia)	NE	**+**	++	++	x
AL Bat Tb	*Tadarida brasiliensis* (Alabama)	DR	++	−	++	x

++ EC_50_ < 1 µg /mL.

+EC_50_ > 1 µg /mL.

− Not neutralized by HuMAb at the highest concentration tested.

x Neutralized by HRIG.

^a^Lab-acquired mutation I338T identified in 3860 Bat following cell culture adaptation. A primary (wild type) isolate of this virus is not available to test.

^b^RAB1 epitope variants with the noted residues at positions 336, and 346.

## Discussion

The standard of care for rabies PEP is a dose of RIG and rabies vaccine given on the day of exposure, followed by a dose of vaccine on days 3, 7, and 14 post-exposure^22–24^. RIG in general is derived from pooled sera of human donors hyper-immunized with rabies vaccine (HRIG) or from hyper-immune horses (ERIG). Timely administration of RIG is critical and lifesaving. Thus, any challenges to the administration of RIG pose a threat to public health^25^. Limited supply, high cost, batch-to-batch variability and theoretical pathogen transmission are the main reasons to seek alternatives such as monoclonal antibodies^26–29^. Although collecting polyclonal sera from immunized donors results in a pool of diverse antibodies targeting numerous neutralizing and non-neutralizing epitopes, advancements in antibody technology make it possible to isolate specific monoclonal antibodies against defined targets on the rabies glycoprotein^30,31^. RAB1 is a fully human antibody developed by our team and licensed by Serum Institute of India (SII) under the trade name RABISHIELD^©^. RABISHIELD^©^ is the first anti-rabies monoclonal antibody product commercially available in the world and is fully licensed for human PEP in India. It was proven to be safe and effective against RABV exposures in India where circulating arctic-like and Indian subcontinent RABV variants are the primary sources of rabies^32,33^.

RAB1 is capable of neutralizing a wide range of terrestrial and bat isolates. RAB1 was mapped to antigenic site III, which is important, because antigenic site III plays a major role in pathogenicity through virus receptor binding and viral dissemination in the nervous system^34,35^. RAB1 escape mutants were found at positions 336 and 346 of antigenic site III. Mutations in both N336 and R346 are required for RAB1 escape. Unlike in India, there are diverse reservoirs of RABV in bats, in addition to terrestrial carnivores, in North America. RAB1 does not fully neutralize all North American bat RABV isolates (Table 1). Therefore, an additional HuMAb is required together with RAB1 in a cocktail for broader neutralization activity. Here we conducted a study to identify and characterize additional HuMAb to be included with RAB1 in a cocktail. We started with the HuMAb panel from which RAB1 was previously chosen, and selected RAB2, which displayed potent neutralization of several RABV strains. The RAB2 epitope is non-overlapping with RAB1 and mapped to antigenic site II. RFFIT testing for RABV isolates against the R172 (RAB1 + RAB2) cocktail revealed isolates with the D33 N336 E346 (DNE) epitope is hard to neutralize at lower concentrations by R172 and requires more antibodies. Notably, higher amounts of the standard of care, HRIG, is also required to neutralize DNE isolates with much lower potency as compared to all other tested variants (Tables 2, 3, 4 and 5). This observation suggests that the HRIG may contain low amount of DNE specific antibodies in its polyclonal mixture of antibodies. In the hamster model, R172 provided equivalent or better protection than HRIG against a canine variant, fox isolate, and bat variant. However, protection against the DNE variants in the hamster model could not be proven due to a lack of mortality when hamsters were challenged with these viruses. These findings led us to a second cocktail of non-overlapping antibodies capable of neutralizing all North American isolates including the DNE variants. CR57 which was previously described by Dietzchold, et al.^21^ as one of the most studied anti-rabies MAb. The CR57 epitope was defined by ELISA binding using a set of small glycoprotein peptides^36^. CR57 epitope was found to be within antigenic site I (a.a.226–231, KLCGVL), among which four residues, K/C/G/V, appeared to be crucial for CR57 binding^36^. Like other anti-rabies MAbs, CR57 does not fully neutralize all RABV isolates including some of the isolates found in North America. Among 38 tested street RABV isolates, CR57 neutralizes 36 of them in RFFIT^12,37^. The two isolates that were not neutralized are south central skunk and the Ef RABV variant from a Myotis sp. (Colorado). We also found that the same south central skunk isolate, which has a lab acquired escape mutation, was not neutralized by CR57. Interestingly, we found that the Ef RABV variant from a Myotis sp. (Colorado) was neutralized, but a Tb variant that was previously reported as neutralized by CR57 escaped neutralization in our study. This would suggest another possible lab acquired mutation in the Alabama bat Tb isolate. However, all isolates currently and previously reported to escape CR57 neutralization are fully neutralized by RAB1 (Tables 4 and 5), or in another words the R173 cocktail.

As emphasized by the WHO and FDA, there is a need for a cocktail of monoclonal antibodies as an alternative to HRIG as a component of PEP for rabies in North America. These guidelines describe in detail the nonclinical and clinical requirements for the development and licensing of monoclonal antibody cocktails in the United States. Our work demonstrated that R173, an antibody cocktail, could neutralize all the viruses that were tested in this study, which are representative of the genetic diversity detected at different loci important for antibody binding. Therefore, the R173 is expected to neutralize all RABV variants in North America including both terrestrial and bat variants tested. R173 is a promising candidate for further clinical development.

## Supplementary Information

Supplementary Figure 1.

Supplementary Table 1.

Supplementary Table 2.

## References

[1] American Academy of Pediatrics CoIDPLKBCJKDWLSSAAoPCoID. Red book : 2012 report of the Committee on Infectious Diseases (2012).

[2] Hampson K, Coudeville L, Lembo T, Sambo M, Kieffer A, Attlan M, Barrat J, Blanton JD, Briggs DJ, Cleaveland S, Costa P, Freuling CM, Hiby E, Knopf L, Leanes F, Meslin FX, Metlin A, Miranda ME, Muller T, Nel LH, Recuenco S, Rupprecht CE, Schumacher C, Taylor L, Vigilato MA, Zinsstag J, Dushoff J, Alliance G (2015). Estimating the global burden of endemic canine rabies. PLoS Negl. Trop. Dis..

[3] Knobel DL, Cleaveland S, Coleman PG, Fevre EM, Meltzer MI, Miranda ME, Shaw A, Zinsstag J, Meslin FX (2005). Re-evaluating the burden of rabies in Africa and Asia. Bull. World Health Organ..

[4] Ma X, Monroe BP, Cleaton JM, Orciari LA, Gigante CM, Kirby JD, Chipman RB, Fehlner-Gardiner C, Gutierrez Cedillo V, Petersen BW, Olson V, Wallace RM (2020). Rabies surveillance in the United States during 2018. J. Am. Vet. Med. Assoc..

[5] Feder HM, Petersen BW, Robertson KL, Rupprecht CE (2012). Rabies: still a uniformly fatal disease? Historical occurrence, epidemiological trends, and paradigm shifts. Curr. Infect. Dis. Rep..

[6] Christian KA, Blanton JD, Auslander M, Rupprecht CE (2009). Epidemiology of rabies post-exposure prophylaxis–United States of America, 2006–2008. Vaccine.

[7] Vora NM, Clippard JR, Stobierski MG, Signs K, Blanton JD (2015). Animal bite and rabies postexposure prophylaxis reporting–United States, 2013. J. Public Health Manag. Pract..

[8] WHO. WHO consultation on a monoclonal antibody cocktail for rabies post exposure treatment (2002).

[9] Sloan SE, Hanlon C, Weldon W, Niezgoda M, Blanton J, Self J, Rowley KJ, Mandell RB, Babcock GJ, Thomas WD, Rupprecht CE, Ambrosino DM (2007). Identification and characterization of a human monoclonal antibody that potently neutralizes a broad panel of rabies virus isolates. Vaccine.

[10] Wang Y, Rowley KJ, Booth BJ, Sloan SE, Ambrosino DM, Babcock GJ (2011). G glycoprotein amino acid residues required for human monoclonal antibody RAB1 neutralization are conserved in rabies virus street isolates. Antiviral. Res..

[11] Pieracci EG, Brown JA, Bergman DL, Gilbert A, Wallace RM, Blanton JD, Velasco-Villa A, Morgan CN, Lindquist S, Chipman RB (2020). Evaluation of species identification and rabies virus characterization among bat rabies cases in the United States. J. Am. Vet. Med. Assoc..

[12] Franka R, Carson WC, Ellison JA, Taylor ST, Smith TG, Kuzmina NA, Kuzmin IV, Marissen WE, Rupprecht CE (2017). In vivo efficacy of a cocktail of human monoclonal antibodies (CL184) against diverse north American bat rabies virus variants. Trop. Med. Infect. Dis..

[13] Anonymous. WHO Expert Consultation on Rabies. Second report. World Health Organ Tech Rep Ser: 1–139, back cover (2013). 24069724

[14] FDA. Rabies: Developing Monoclonal Antibody Cocktails for the Passive Immunization Component of Post-Exposure Prophylaxis, Guidance for Industry (2021).

[15] Préhaud C, Lay S, Dietzschold B, Lafon M (2003). Glycoprotein of nonpathogenic rabies viruses is a key determinant of human cell apoptosis. J. Virol..

[16] He J, Choe S, Walker R, Di Marzio P, Morgan DO, Landau NR (1995). Human immunodeficiency virus type 1 viral protein R (Vpr) arrests cells in the G2 phase of the cell cycle by inhibiting p34cdc2 activity. J. Virol..

[17] Connor RI, Chen BK, Choe S, Landau NR (1995). Vpr is required for efficient replication of human immunodeficiency virus type-1 in mononuclear phagocytes. Virology.

[18] Yager ML, Moore SM, Rupprecht C, Nagarajan T (2015). The Rapid Fluorescent Focus Inhibition Test. Current laboratory techniques in rabies diagnosis, research and prevention.

[19] Dean DJ, Abelseth MK, Atanasiu P, Meslin FX, Kaplan MM, Koprowski H (1996). The fluorescent antibody test. Laboratory techniques in rabies.

[20] Wadhwa A, Wilkins K, Gao J, Condori Condori RE, Gigante CM, Zhao H, Ma X, Ellison JA, Greenberg L, Velasco-Villa A, Orciari L, Li Y (2017). A pan-lyssavirus taqman real-time RT-PCR assay for the detection of highly variable rabies virus and other lyssaviruses. PLoS Negl. Trop. Dis..

[21] Dietzschold B, Gore M, Casali P, Ueki Y, Rupprecht CE, Notkins AL, Koprowski H (1990). Biological characterization of human monoclonal antibodies to rabies virus. J. Virol..

[22] Zhu S, Guo C (2016). Rabies control and treatment: from prophylaxis to strategies with curative potential. Viruses.

[23] Brunker K, Mollentze N (2018). Rabies virus. Trends Microbiol..

[24] Tarantola A, Tejiokem MC, Briggs DJ (2019). Evaluating new rabies post-exposure prophylaxis (PEP) regimens or vaccines. Vaccine.

[25] Reid-Sanden FL, Fishbein DB, Stevens CA, Briggs DJ (1991). Administration of rabies vaccine in the gluteal area: A continuing problem. Arch. Intern. Med..

[26] Ziesenitz VC, Mazer-Amirshahi M, Zocchi MS, Fox ER, May LS (2017). U.S. vaccine and immune globulin product shortages, 2001–15. Am. J. Health Syst. Pharm..

[27] Abreu-Mota T, Hagen KR, Cooper K, Jahrling PB, Tan G, Wirblich C, Johnson RF, Schnell MJ (2018). Non-neutralizing antibodies elicited by recombinant Lassa-Rabies vaccine are critical for protection against Lassa fever. Nat. Commun..

[28] Dh AN, Haradanhalli RS (2019). Assessment of procurement, distribution, availability, and utilization of rabies biologicals for postexposure prophylaxis in seven states of India. Indian J. Public Health.

[29] Bharti OK, Thakur B, Rao R (2019). Wound-only injection of rabies immunoglobulin (RIG) saves lives and costs less than a dollar per patient by "pooling strategy". Vaccine.

[30] Hobart-Porter N, Stein M, Toh N, Amega N, Nguyen HB, Linakis J (2021). Safety and efficacy of rabies immunoglobulin in pediatric patients with suspected exposure. Hum. Vaccin. Immunother.

[31] Nagarajan T, Rupprecht CE, Dessain SK, Rangarajan PN, Thiagarajan D, Srinivasan VA (2008). Human monoclonal antibody and vaccine approaches to prevent human rabies. Curr. Top. Microbiol. Immunol..

[32] Gill GS, Singh BB, Dhand NK, Aulakh RS, Sandhu BS, Ward MP, Brookes VJ (2019). Estimation of the incidence of animal rabies in Punjab India. PLoS ONE.

[33] Gigante CM, Yale G, Condori RE, Costa NC, Long NV, Minh PQ, Chuong VD, Tho ND, Thanh NT, Thin NX, Hanh NTH, Wambura G, Ade F, Mito O, Chuchu V, Muturi M, Mwatondo A, Hampson K, Thumbi SM, Thomae BG, de Paz VH, Meneses S, Munyua P, Moran D, Cadena L, Gibson A, Wallace RM, Pieracci EG, Li Y (2020). Portable rabies virus sequencing in canine rabies endemic countries using the Oxford Nanopore MinION. Viruses.

[34] Dietzschold B, Wunner WH, Wiktor TJ, Lopes AD, Lafon M, Smith CL, Koprowski H (1983). Characterization of an antigenic determinant of the glycoprotein that correlates with pathogenicity of rabies virus. Proc. Natl. Acad. Sci. USA.

[35] Diallo A (1986). Avirulent mutants of the rabies virus: change in site III of the glycoprotein. Ann. Rech. Vet..

[36] Marissen WE, Kramer RA, Rice A, Weldon WC, Niezgoda M, Faber M, Slootstra JW, Meloen RH, Clijsters-van der Horst M, Visser TJ, Jongeneelen M, Thijsse S, Throsby M, de Kruif J, Rupprecht CE, Dietzschold B, Goudsmit J, Bakker ABH (2005). Novel rabies virus-neutralizing epitope recognized by human monoclonal antibody: Fine mapping and escape mutant analysis. J. Virol..

[37] Bakker AB, Marissen WE, Kramer RA, Rice AB, Weldon WC, Niezgoda M, Hanlon CA, Thijsse S, Backus HH, de Kruif J, Dietzschold B, Rupprecht CE, Goudsmit J (2005). Novel human monoclonal antibody combination effectively neutralizing natural rabies virus variants and individual in vitro escape mutants. J. Virol..

