# Supplementary material for: A cocktail of human monoclonal antibodies broadly neutralizes North American rabies virus variants as a promising candidate for rabies post-exposure prophylaxis
Source: Sci Rep. 2022 Jun 7;12:9403. doi: 10.1038/s41598-022-13527-0 (PMC9174473; doi:10.1038/s41598-022-13527-0)
Supplement: Supplementary file 1 — Supplementary Figure 1. [file 41598_2022_13527_MOESM1_ESM.docx]

**Supplementary Figure 1**. **Efficacy of R172 (1:1 cocktail of RAB1 and RAB2) in the Syrian hamster rabies PEP model**. (Top) Diagram of the experimental design. Hamsters were infected with rabies virus and 24 hours later PEP was initiated with 20 IU/mL HRIG, 1.1 mg/kg of an irrelevant HuMAb, 1.1 mg/kg of R172, 0.1 mg/kg of R172, 0.01 mg/kg of R172, or no PEP depending on the group. All groups receiving PEP also received human rabies vaccine (Immovax) on days 1, 4, 15 and 29 post-infection (days 0, 3, 14, 28 of PEP). Animals were monitored daily, twice daily from day 7-21 post-infection, and euthanized at the first clinical signs of rabies. All animals surviving to endpoint (day 45 post-infection) were euthanized. **A**. Kaplan-Meier survival curve for hamsters infected with Bat EF variant. **B**. Kaplan-Meier survival curve for hamsters infected with Texas coyote 323R isolate, cosmopolitan dog RABV variant. **C**. Kaplan-Meier survival curve for hamsters infected with Arizona fox 2400 variant. Figure was created with BioRender.com

**
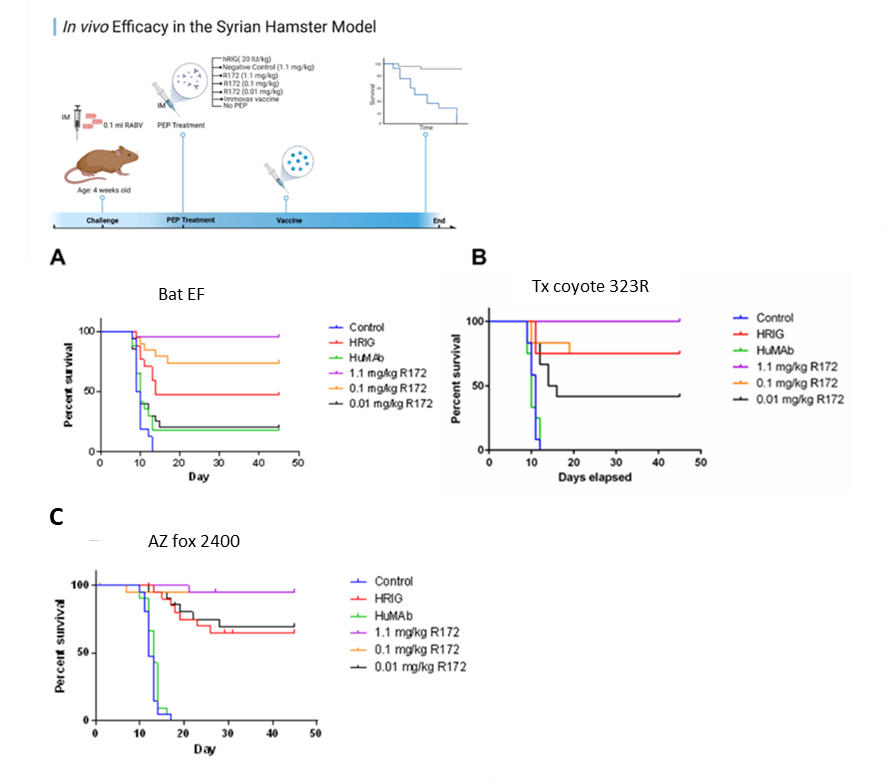
**
