# Supplementary material for: A cocktail of human monoclonal antibodies broadly neutralizes North American rabies virus variants as a promising candidate for rabies post-exposure prophylaxis
Source: Sci Rep. 2022 Jun 7;12:9403. doi: 10.1038/s41598-022-13527-0 (PMC9174473; doi:10.1038/s41598-022-13527-0)
Supplement: Supplementary file 2 — Supplementary Table 1. [file 41598_2022_13527_MOESM2_ESM.docx]

**Table S1. 50% effective concentration (EC_50_) in µg/mL from RFFIT**

| **Viral Isolate** | **Source (location)** | **Epitope** | **RAB1** | **RAB2** | **R172** | **CR57** | **R173** |
| --- | --- | --- | --- | --- | --- | --- | --- |
| TX Coyote 323R | Coyote (Texas) | ENR | 0.01 | 2 | 0.01 | ND ^a^ | ND |
| AK Fox | Fox (Alaska) | ENR | 0.003 | 0.004 | 0.002 | ND | ND |
| CA SK | Skunk (California) | ENR | 0.008 | 0.5 | 0.01 | ND | ND |
| PR Mong | Mongoose (Puerto Rico) | ENR | 0.006 | 0.008 | 0.006 | ND | ND |
| Sonora Dog | Dog (Mexico) | ENR | 0.004 | 0.004 | 0.003 | ND | ND |
| TXSK 4380 ^b^ | Skunk (Texas) | ENR | 0.004 | 0.01 | 0.005 | 0.006 wt | 0.01 |
|  |  |  |  |  |  | >1000 |  |
| TXSK 5171 ^b^ | Skunk (Texas) | ENR | 0.005 wt | 0.008 | 0.02 | 0.007 | 0.02 |
|  |  |  | >1000 |  |  |  |  |
| ERA | Lab Strain | ENR | 0.001 | 0.002 | 0.001 | ND | ND |
| NCSK | Skunk (North-central US) | ENK | 0.7 | 0.004 | 0.008 | ND | ND |
| TXFX | Fox (Texas) | ENK | 0.02 | 0.02 | 0.01 | ND | ND |
| RAC | Raccoon (Southeast US) | ESR | 0.01 | 0.03 | 0.007 | ND | ND |
| CVS-11 | Lab Strain | EDR | 3 | 0.01 | 0.003 | 0.006 | 0.01 |
| DR Brazil | *Desmodus rotundus* (Brazil) | ENR | 0.2 | 0.002 | 0.002 | ND | ND |
| C1434 | *Perimyotis subflavus* (Alabama) | ENK | 0.6 | 0.01 | 0.01 | 0.1 | 0.2 |
| CO EF Bat 6938 | Ef variant in a *Myotis* sp. (Colorado) | ENK | 0.07 | 0.008 | 0.01 | 0.4 | 0.03 |
| WA Bat | *Lasionycteris noctivagans* (Washington) | ENK | 0.1 | 0.006 | 0.005 | 0.6 | 0.04 |
| AZ Fox 2400 | Ef variant in a fox (Arizona) | ENK | 0.2 | 0.04 | 0.1 | ND | ND |
| AZ 3860 Bat ^c^ | *Parastrellus hesperus* (Arizona) | ENS | >2000 | 0.008 | 0.01 | 0.008 | 0.04 |
| CA Bat | *Myotis thysanodes* (California) | ENS | 0.006 | 0.004 | 0.003 | ND | ND |
| Myotis | *Myotis* sp.(Washington) | ENS | 0.003 | 0.006 | 0.004 | ND | ND |
| 1625 Bat | *Lasiurus borealis* (Michigan) | DNE | 30 | 50 | 10 | 0.04 | 0.07 |
| AZ Bat LC | *Aeorestes cinereus* (Arizona) | DNE | 10 | 400 | 20 | 0.04 | 0.07 |
| FL Bat 769 | *Lasiurus seminolus* (Florida) | DNE | 80 | >2000 | 400 | 0.1 | 0.3 |
| TN132 | *Lasiurus borealis* (Tennessee) | DNE | 2000 | >2000 | 1000 | 0.01 | 0.02 |
| TN269 | *Lasiurus borealis* (Tennessee) | DNE | 1000 | >2000 | 2000 | 0.03 | 0.04 |
| TN33 | *Lasiurus borealis* (Tennessee) | DNE | 9 | 1000 | 5 | 0.008 | 0.03 |
| TN410 | *Aeorestes cinereus* (Tennessee) | DNE | 4 | 80 | 6 | 0.1 | 0.07 |
| VA1340 | *Lasiurus borealis* (Virginia) | DNE | 200 | >2000 | 200 | 0.08 | 0.2 |
| VA399 | *Lasiurus borealis* (Virginia) | DNE | 20 | 900 | 40 | 0.04 | 0.1 |
| Bat EF | *Eptesicus fuscus* (Pennsylvania) | EDR | 0.2 | 0.009 | 0.02 | ND | ND |
| AL Bat Tb | *Tadarida brasiliensis* (Alabama) | DR | 0.2 | ND | ND | >1000 | 0.08 |

^a^ Not determined (ND)

^b^ Lab-acquired mutations following additional cell culture passage. Wild-type (wt) primary isolate was tested.

^c^ Lab-acquired mutation I338T identified in 3860 Bat following cell culture adaptation. A primary (wild-type) isolate of this virus is not available to test.
