# Supplementary material for: A cocktail of human monoclonal antibodies broadly neutralizes North American rabies virus variants as a promising candidate for rabies post-exposure prophylaxis
Source: Sci Rep. 2022 Jun 7;12:9403. doi: 10.1038/s41598-022-13527-0 (PMC9174473; doi:10.1038/s41598-022-13527-0)
Supplement: Supplementary file 3 — Supplementary Table 2. [file 41598_2022_13527_MOESM3_ESM.docx]

**Table S2. North American rabies virus isolates**

| **GenBank ID#** | **Protein ID#** | **RAB1 (N336)** | **RAB1 (K346)** | **RAB2 (E33)** | **Origin** | **Continent** | **Animal Source** |
| --- | --- | --- | --- | --- | --- | --- | --- |
| JQ685978 | AFN24505 | N336 | S346 | E33 | USA | North America | Gray fox |
| JQ685979 | AFN24506 | N336 | S346 | E33 | USA | North America | Gray fox |
| JQ685980 | AFN24507 | N336 | R346 | E33 | USA | North America | Domestic dog |
| JQ685984 | AFN24511 | N336 | R346 | E33 | USA | North America | Gray fox |
| JQ685985 | AFN24512 | N336 | R346 | E33 | USA | North America | Gray fox |
| JQ685986 | AFN24513 | N336 | R346 | E33 | USA | North America | striped skunk |
| JQ685987 | AFN24514 | N336 | R346 | E33 | USA | North America | Red fox |
| JQ685988 | AFN24515 | N336 | R346 | E33 | USA | North America | Red fox |
| JQ685989 | AFN24516 | N336 | R346 | E33 | USA | North America | Gray fox |
| JQ685990 | AFN24517 | N336 | R346 | E33 | USA | North America | Red fox |
| JQ685991 | AFN24518 | N336 | R346 | E33 | USA | North America | Wolf |
| JQ685993 | AFN24520 | N336 | R346 | E33 | USA | North America | Felis catus |
| JQ685994 | AFN24521 | S336 | R346 | E33 | USA | North America | Coyote |
| JQ685995 | AFN24522 | N336 | R346 | E33 | USA | North America | Gray fox |
| JQ685996 | AFN24523 | N336 | K346 | E33 | USA | North America | Big brown bat |
| JQ685997 | AFN24524 | N336 | S346 | E33 | USA | North America | Myotis sp (bat) |
| JQ685998 | AFN24525 | N336 | S346 | E33 | USA | North America | Myotis sp (bat) |
| JQ685999 | AFN24526 | N336 | E346 | D33 | USA | North America | Lasiurus cinereus (bat) |
| JQ686000 | AFN24527 | N336 | K346 | E33 | USA | North America | Perimyotis subflavus (bat) |
| JQ686001 | AFN24528 | N336 | K346 | E33 | USA | North America | Lasionycteris noctivagans (bat) |
| JQ686002 | AFN24529 | D336 | R346 | E33 | USA | North America | Eptesicus fuscus (bat) |
| JQ686003 | AFN24530 | N336 | K346 | E33 | USA | North America | Perimyotis subflavus (bat) |
| JQ686004 | AFN24531 | N336 | K346 | E33 | USA | North America | Lasionycteris noctivagans (bat) |
| JQ686005 | AFN24532 | N336 | K346 | E33 | USA | North America | Lasiurus intermedius (bat) |
| JQ686006 | AFN24533 | N336 | S346 | E33 | USA | North America | Antrozous pallidus (bat) |
| JQ686007 | AFN24534 | N336 | E346 | D33 | USA | North America | Lasiurus borealis (bat) |
| JQ686008 | AFN24535 | N336 | K346 | E33 | USA | North America | Gray fox |
| JQ686009 | AFN24536 | N336 | K346 | E33 | USA | North America | Red fox |
| JQ686010 | AFN24537 | D336 | R346 | E33 | USA | North America | fox |
| KC791791 | AGN94067 | N336 | K346 | E33 | Mexico | North America | Desmodus rotundus (bat) |
| KC791795 | AGN94071 | N336 | R346 | E33 | Mexico | North America | Skunk |
| KC791796 | AGN94072 | N336 | R346 | E33 | Mexico | North America | Skunk |
| KC791797 | AGN94073 | N336 | K346 | E33 | Mexico | North America | Horse |
| KC791798 | AGN94312 | N336 | K346 | E33 | USA | North America | bat |
| KC791799 | AGN94075 | N336 | R346 | E33 | Mexico | North America | cow |
| KC791801 | AGN94077 | N336 | E346 | D33 | USA | North America | bat |
| KC791803 | AGN94079 | N336 | K346 | E33 | USA | North America | puma |
| KC791804 | AGN94080 | N336 | K346 | E33 | Mexico | North America | bat |
| KC791805 | AGN94081 | N336 | K346 | E33 | USA | North America | bat |
| KC791806 | AGN94082 | S336 | R346 | E33 | USA | North America | Gray fox |
| KC791807 | AGN94083 | N336 | K346 | E33 | USA | North America | Skunk |
| KC791808 | AGN94084 | N336 | R346 | E33 | USA | North America | Cow |
| KC791809 | AGN94085 | N336 | R346 | E33 | Mexico | North America | Skunk |
| KC791810 | AGN94086 | N336 | R346 | E33 | USA | North America | Bobcat |
| KC791811 | AGN94087 | N336 | K346 | E33 | Mexico | North America | Cow |
| KC791812 | AGN94088 | N336 | R346 | E33 | USA | North America | Raccoon |
| KC791813 | AGN94089 | S336 | R346 | E33 | USA | North America | Beaver |
| KC791814 | AGN94090 | N336 | R346 | E33 | USA | North America | Dog |
| KC791815 | AGN94091 | N336 | R346 | E33 | USA | North America | Red fox |
| KC791816 | AGN94092 | N336 | R346 | E33 | USA | North America | Red fox |
| KC791817 | AGN94093 | N336 | K346 | E33 | USA | North America | striped skunk |
| KC791818 | AGN94094 | N336 | R346 | E33 | USA | North America | Coyote |
| KC791819 | AGN94095 | N336 | R346 | E33 | USA | North America | Gray fox |
| KC791820 | AGN94096 | N336 | R346 | E33 | USA | North America | Gray fox |
| KC791821 | AGN94097 | N336 | R346 | E33 | USA | North America | Gray fox |
| KC791822 | AGN94098 | N336 | R346 | E33 | USA | North America | Gray fox |
| KC791823 | AGN94099 | N336 | R346 | E33 | USA | North America | Coyote |
| KC791824 | AGN94100 | N336 | R346 | E33 | USA | North America | Lynx rufus |
| KC791825 | AGN94101 | N336 | R346 | E33 | USA | North America | Gray fox |
| KC791826 | AGN94102 | N336 | R346 | E33 | USA | North America | Skunk |
| KC791827 | AGN94103 | N336 | R346 | E33 | USA | North America | Skunk |
| KC791828 | AGN94104 | N336 | R346 | E33 | USA | North America | Skunk |
| KC791829 | AGN94105 | N336 | R346 | E33 | USA | North America | Skunk |
| KC791830 | AGN94106 | N336 | R346 | E33 | USA | North America | Skunk |
| KC791831 | AGN94107 | N336 | R346 | E33 | USA | North America | Skunk |
| KC791832 | AGN94108 | N336 | R346 | E33 | USA | North America | Skunk |
| KC791833 | AGN94109 | N336 | R346 | E33 | USA | North America | Skunk |
| KC791834 | AGN94110 | N336 | R346 | E33 | USA | North America | Skunk |
| KC791835 | AGN94111 | N336 | R346 | E33 | USA | North America | Skunk |
| KC791836 | AGN94112 | N336 | R346 | E33 | USA | North America | Skunk |
| KC791837 | AGN94113 | N336 | R346 | E33 | USA | North America | Skunk |
| KC791838 | AGN94114 | N336 | R346 | E33 | USA | North America | Skunk |
| KC791839 | AGN94115 | N336 | R346 | E33 | USA | North America | Skunk |
| KC791840 | AGN94116 | N336 | R346 | E33 | USA | North America | Skunk |
| KC791841 | AGN94117 | N336 | R346 | E33 | USA | North America | Skunk |
| KC791842 | AGN94118 | N336 | R346 | E33 | USA | North America | Skunk |
| KC791843 | AGN94119 | N336 | R346 | E33 | USA | North America | Skunk |
| KC791844 | AGN94120 | N336 | R346 | E33 | USA | North America | Skunk |
| KC791845 | AGN94121 | N336 | R346 | E33 | USA | North America | Skunk |
| KC791846 | AGN94122 | N336 | R346 | E33 | USA | North America | Skunk |
| KC791847 | AGN94123 | N336 | R346 | E33 | USA | North America | Skunk |
| KC791848 | AGN94124 | N336 | R346 | E33 | USA | North America | Homo sapiens |
| KC791849 | AGN94125 | N336 | K346 | E33 | USA | North America | Gray fox |
| KC791850 | AGN94126 | N336 | R346 | E33 | USA | North America | Lynx rufus |
| KC791851 | AGN94127 | D336 | R346 | E33 | USA | North America | Eptesicus fuscus (bat) |
| KC791854 | AGN94130 | N336 | R346 | E33 | USA | North America | Red fox |
| KC791855 | AGN94131 | D336 | R346 | E33 | USA | North America | Eptesicus fuscus (bat) |
| KC791856 | AGN94132 | N336 | K346 | E33 | USA | North America | Tadarida brasiliensis (bat) |
| KC791857 | AGN94133 | N336 | E346 | D33 | USA | North America | Lasiurus xanthinus (bat) |
| KC791858 | AGN94134 | N336 | S346 | E33 | USA | North America | Parastrellus Hesperus (bat) |
| KC791859 | AGN94135 | N336 | K346 | E33 | USA | North America | Tadarida brasiliensis (bat) |
| KC791860 | AGN94136 | N336 | S346 | E33 | USA | North America | Parastrellus Hesperus (bat) |
| KC791861 | AGN94137 | N336 | R346 | E33 | USA | North America | Red fox |
| KC791862 | AGN94138 | S336 | R346 | E33 | USA | North America | Cat |
| KC791863 | AGN94139 | S336 | R346 | E33 | USA | North America | Raccoon |
| KC791864 | AGN94140 | S336 | R346 | E33 | USA | North America | Raccoon |
| KC791865 | AGN94141 | S336 | R346 | E33 | USA | North America | Raccoon |
| KC791866 | AGN94142 | S336 | R346 | E33 | USA | North America | Raccoon |
| KC791867 | AGN94143 | N336 | R346 | E33 | USA | North America | A11_0625 |
| KC791868 | AGN94144 | N336 | R346 | E33 | USA | North America | A11_0626 |
| KC791869 | AGN94145 | N336 | R346 | E33 | USA | North America | A11_0626 |
| KC791870 | AGN94146 | N336 | R346 | E33 | USA | North America | A11_0627 |
| KC791871 | AGN94147 | S336 | R346 | E33 | USA | North America | A11_0628 |
| KC791872 | AGN94148 | S336 | R346 | E33 | USA | North America | Cat |
| KC791873 | AGN94149 | S336 | R346 | E33 | USA | North America | Raccoon |
| KC791874 | AGN94150 | S336 | R346 | E33 | USA | North America | Raccoon |
| KC791875 | AGN94151 | S336 | R346 | E33 | USA | North America | Raccoon |
| KC791876 | AGN94152 | S336 | R346 | E33 | USA | North America | Cat |
| KC791877 | AGN94153 | N336 | R346 | E33 | USA | North America | A11_1197 |
| KC791878 | AGN94154 | N336 | R346 | E33 | USA | North America | A11_1232 |
| KC791879 | AGN94155 | N336 | R346 | E33 | USA | North America | A11_1233 |
| KC791880 | AGN94156 | N336 | R346 | E33 | USA | North America | A11_1234 |
| KC791881 | AGN94157 | N336 | R346 | E33 | USA | North America | A11_1235 |
| KC791882 | AIJ01128 | N336 | K346 | E33 | USA | North America | Eptesicus fuscus (bat) |
| KC791883 | AGN94159 | N336 | K346 | E33 | USA | North America | Eptesicus fuscus (bat) |
| KC791884 | AGN94160 | N336 | K346 | E33 | USA | North America | Eptesicus fuscus (bat) |
| KC791885 | AGN94161 | N336 | K346 | E33 | USA | North America | Eptesicus fuscus (bat) |
| KC791886 | AGN94162 | N336 | K346 | E33 | USA | North America | A11_1266 |
| KC791887 | AGN94163 | N336 | R346 | E33 | USA | North America | A11_1267 |
| KC791888 | AGN94164 | N336 | R346 | E33 | USA | North America | Arctic fox |
| KC791889 | AGN94165 | N336 | R346 | E33 | USA | North America | Arctic fox |
| KC791890 | AGN94166 | N336 | R346 | E33 | USA | North America | A11_1270 |
| KC791891 | AGN94167 | N336 | R346 | E33 | USA | North America | Red fox |
| KC791892 | AGN94168 | N336 | K346 | E33 | USA | North America | Eptesicus fuscus (bat) |
| KC791893 | AGN94169 | N336 | K346 | E33 | USA | North America | Eptesicus fuscus (bat) |
| KC791894 | AGN94170 | N336 | K346 | E33 | USA | North America | Eptesicus fuscus (bat) |
| KC791895 | AGN94171 | N336 | K346 | E33 | USA | North America | Eptesicus fuscus (bat) |
| KC791896 | AGN94172 | N336 | K346 | E33 | USA | North America | A11_1301 |
| KC791897 | AGN94173 | N336 | R346 | E33 | USA | North America | Skunk |
| KC791898 | AGN94174 | N336 | R346 | E33 | USA | North America | A11_1642 |
| KC791899 | AGN94175 | N336 | R346 | E33 | USA | North America | Skunk |
| KC791900 | AGN94176 | N336 | R346 | E33 | USA | North America | A11_1644 |
| KC791901 | AGN94177 | N336 | R346 | E33 | USA | North America | Skunk |
| KC791902 | AGN94178 | N336 | R346 | E33 | USA | North America | Skunk |
| KC791903 | AGN94179 | N336 | R346 | E33 | USA | North America | Skunk |
| KC791904 | AGN94180 | N336 | R346 | E33 | USA | North America | Skunk |
| KC791906 | AGN94182 | S336 | R346 | E33 | USA | North America | Cat |
| KC791907 | AGN94183 | S336 | R346 | E33 | USA | North America | Raccoon |
| KC791908 | AGN94184 | S336 | R346 | E33 | USA | North America | Raccoon |
| KC791909 | AGN94185 | S336 | R346 | E33 | USA | North America | Raccoon |
| KC791910 | AGN94186 | N336 | R346 | E33 | USA | North America | Skunk |
| KC791911 | AGN94187 | N336 | R346 | E33 | USA | North America | Skunk |
| KC791912 | AGN94188 | N336 | R346 | E33 | USA | North America | Skunk |
| KC791913 | AGN94189 | N336 | R346 | E33 | USA | North America | Skunk |
| KC791914 | AGN94190 | N336 | R346 | E33 | USA | North America | Lynx rufus |
| KC791915 | AGN94191 | N336 | K346 | E33 | USA | North America | Skunk |
| KC791916 | AGN94192 | N336 | R346 | E33 | USA | North America | Skunk |
| KC791917 | AGN94193 | N336 | R346 | E33 | USA | North America | Skunk |
| KC791918 | AGN94194 | N336 | R346 | E33 | USA | North America | Skunk |
| KC791919 | AGN94195 | N336 | R346 | E33 | USA | North America | Skunk |
| KC791920 | AGN94196 | N336 | R346 | E33 | USA | North America | Skunk |
| KC791921 | AGN94197 | N336 | R346 | E33 | USA | North America | Skunk |
| KC791922 | AGN94198 | N336 | R346 | E33 | USA | North America | Skunk |
| KC791923 | AGN94199 | N336 | R346 | E33 | USA | North America | Skunk |
| KC791924 | AGN94200 | N336 | R346 | E33 | USA | North America | Skunk |
| KC791925 | AGN94201 | N336 | R346 | E33 | USA | North America | Skunk |
| KC791926 | AGN94202 | N336 | R346 | E33 | USA | North America | Skunk |
| KC791927 | AGN94203 | N336 | R346 | E33 | USA | North America | Skunk |
| KC791928 | AGN94204 | N336 | R346 | E33 | USA | North America | Skunk |
| KC791929 | AGN94205 | N336 | R346 | E33 | USA | North America | Skunk |
| KC791930 | AGN94206 | N336 | R346 | E33 | USA | North America | Skunk |
| KC791931 | AGN94207 | N336 | R346 | E33 | USA | North America | Skunk |
| KC791932 | AGN94208 | N336 | R346 | E33 | USA | North America | Skunk |
| KC791933 | AGN94209 | N336 | R346 | E33 | USA | North America | Skunk |
| KC791934 | AGN94210 | N336 | R346 | E33 | USA | North America | Skunk |
| KC791935 | AGN94211 | N336 | R346 | E33 | USA | North America | Skunk |
| KC791936 | AGN94212 | N336 | R346 | E33 | USA | North America | Skunk |
| KC791937 | AGN94213 | N336 | R346 | E33 | USA | North America | Skunk |
| KC791938 | AGN94214 | N336 | R346 | E33 | USA | North America | Skunk |
| KC791939 | AGN94215 | N336 | R346 | E33 | USA | North America | Skunk |
| KC791940 | AGN94216 | N336 | R346 | E33 | USA | North America | Skunk |
| KC791941 | AGN94217 | N336 | R346 | E33 | USA | North America | Skunk |
| KC791942 | AGN94218 | N336 | R346 | E33 | USA | North America | Skunk |
| KC791943 | AGN94219 | N336 | R346 | E33 | USA | North America | Skunk |
| KC791944 | AGN94220 | N336 | R346 | E33 | USA | North America | Skunk |
| KC791945 | AGN94221 | N336 | R346 | E33 | USA | North America | Skunk |
| KC791946 | AGN94222 | N336 | R346 | E33 | USA | North America | Skunk |
| KC791947 | AGN94223 | N336 | R346 | E33 | USA | North America | Skunk |
| KC791948 | AGN94224 | N336 | R346 | E33 | USA | North America | Skunk |
| KC791949 | AGN94225 | N336 | R346 | E33 | USA | North America | Skunk |
| KC791950 | AGN94226 | N336 | R346 | E33 | USA | North America | Skunk |
| KC791951 | AGN94227 | N336 | R346 | E33 | USA | North America | Skunk |
| KC791952 | AGN94228 | N336 | R346 | E33 | USA | North America | Skunk |
| KC791953 | AGN94229 | N336 | R346 | E33 | USA | North America | Skunk |
| KC791954 | AGN94230 | N336 | R346 | E33 | USA | North America | Skunk |
| KC791955 | AGN94231 | N336 | K346 | E33 | USA | North America | Skunk |
| KC791956 | AGN94232 | N336 | R346 | E33 | USA | North America | Lynx rufus |
| KC791957 | AGN94233 | S336 | R346 | E33 | USA | North America | Cat |
| KC791958 | AGN94234 | S336 | R346 | E33 | USA | North America | Cat |
| KC791959 | AGN94235 | S336 | R346 | E33 | USA | North America | Cat |
| KC791960 | AGN94236 | S336 | R346 | E33 | USA | North America | Marmota monax |
| KC791961 | AGN94237 | N336 | R346 | E33 | Mexico | North America | Coyote |
| KC791962 | AGN94238 | N336 | R346 | E33 | USA | North America | Homo sapiens |
| KC791963 | AGN94239 | N336 | K346 | E33 | USA | North America | Perimyotis subflavus (bat) |
| KC791964 | AGN94240 | N336 | E346 | D33 | USA | North America | Lasiurus borealis (bat) |
| KC791965 | AGN94241 | S336 | R346 | E33 | USA | North America | Skunk |
| KC791966 | AGN94242 | N336 | K346 | E33 | USA | North America | Perimyotis subflavus (bat) |
| KC791967 | AGN94243 | N336 | K346 | E33 | USA | North America | Eptesicus fuscus (bat) |
| KC791968 | AGN94244 | N336 | R346 | E33 | USA | North America | Skunk |
| KC791969 | AGN94245 | N336 | R346 | E33 | USA | North America | Skunk |
| KC791970 | AGN94246 | N336 | E346 | D33 | USA | North America | Lasiurus borealis (bat) |
| KC791971 | AGN94247 | N336 | E346 | D33 | USA | North America | Lasiurus borealis (bat) |
| KC791986 | AGN94262 | N336 | R346 | E33 | USA | North America | Cow |
| KC792001 | AGN94277 | S336 | R346 | E33 | USA | North America | Cat |
| KC792002 | AGN94278 | S336 | R346 | E33 | USA | North America | Cat |
| KC792003 | AGN94279 | S336 | R346 | E33 | USA | North America | Cat |
| KC792004 | AGN94280 | S336 | R346 | E33 | USA | North America | Cat |
| KC792005 | AGN94281 | N336 | E346 | D33 | USA | North America | Lasiurus borealis (bat) |
| KC792006 | AGN94282 | S336 | R346 | E33 | USA | North America | Raccoon |
| KC792007 | AGN94283 | S336 | R346 | E33 | USA | North America | Dog |
| KC792008 | AGN94284 | N336 | R346 | E33 | USA | North America | Skunk |
| KC792009 | AGN94285 | N336 | R346 | E33 | USA | North America | Skunk |
| KC792010 | AGN94286 | N336 | R346 | E33 | USA | North America | Skunk |
| KC792011 | AGN94287 | N336 | R346 | E33 | USA | North America | Skunk |
| KC792012 | AGN94288 | N336 | R346 | E33 | USA | North America | Skunk |
| KC792013 | AGN94289 | N336 | R346 | E33 | USA | North America | Skunk |
| KC792014 | AGN94290 | N336 | R346 | E33 | USA | North America | Skunk |
| KC792015 | AGN94291 | N336 | R346 | E33 | USA | North America | Skunk |
| KC792016 | AGN94292 | N336 | R346 | E33 | USA | North America | Skunk |
| KC792017 | AGN94293 | N336 | K346 | E33 | USA | North America | Gray fox |
| KC792018 | AGN94294 | N336 | R346 | E33 | USA | North America | Pecari tajacu |
| KC792019 | AGN94295 | G336 | R346 | E33 | USA | North America | Cat |
| KC792020 | AGN94296 | S336 | R346 | E33 | USA | North America | Cat |
| KC792021 | AGN94297 | S336 | R346 | E33 | USA | North America | Cat |
| KC792022 | AGN94298 | S336 | R346 | E33 | USA | North America | Marmota monax |
| KC792023 | AGN94299 | S336 | R346 | E33 | USA | North America | Cat |
| KC792024 | AGN94300 | N336 | R346 | E33 | USA | North America | Skunk |
| KC792025 | AGN94301 | N336 | R346 | E33 | USA | North America | Skunk |
| KC792026 | AGN94302 | N336 | K346 | E33 | USA | North America | Skunk |
| KC792027 | AGN94303 | G336 | R346 | E33 | USA | North America | Skunk |
| KC792028 | AGN94304 | S336 | R346 | E33 | USA | North America | Fox |
| KC792029 | AGN94305 | S336 | R346 | E33 | USA | North America | Cat |
| KC792030 | AGN94306 | S336 | R346 | E33 | USA | North America | Cat |
| KC792031 | AGN94307 | G336 | R346 | E33 | USA | North America | Cat |
| KC792033 | AGN94309 | N336 | R346 | E33 | USA | North America | Fox |
| KC792034 | AGN94310 | S336 | R346 | E33 | USA | North America | Cat |
| KC792035 | AGN94311 | S336 | R346 | E33 | USA | North America | Dog |
| KC792036 | AGN94074 | N336 | K346 | E33 | USA | North America | Skunk |
| KC792037 | AGN94313 | N336 | R346 | E33 | USA | North America | Skunk |
| KC792038 | AGN94314 | N336 | R346 | E33 | USA | North America | Skunk |
| KC792039 | AGN94315 | N336 | R346 | E33 | USA | North America | Skunk |
| KC792040 | AGN94316 | S336 | R346 | E33 | USA | North America | Cat |
| KC792041 | AGN94317 | S336 | R346 | E33 | USA | North America | Cat |
| KC792042 | AGN94318 | S336 | R346 | E33 | USA | North America | Cat |
| KC792043 | AGN94319 | N336 | R346 | E33 | USA | North America | Gray fox |
| KC792044 | AGN94320 | N336 | K346 | E33 | USA | North America | Eptesicus fuscus (bat) |
| KC792045 | AGN94321 | S336 | R346 | E33 | USA | North America | Cat |
| KC792046 | AGN94322 | S336 | R346 | E33 | USA | North America | Cat |
| KC792047 | AGN94323 | S336 | R346 | E33 | USA | North America | Cat |
| KC792048 | AGN94324 | S336 | R346 | E33 | USA | North America | Cat |
| KC792049 | AGN94325 | N336 | K346 | E33 | USA | North America | Gray fox |
| KC792050 | AGN94326 | S336 | R346 | E33 | USA | North America | Cat |
| KC792052 | AGN94328 | N336 | K346 | E33 | USA | North America | Bat |
| KC792053 | AGN94329 | N336 | R346 | E33 | USA | North America | Skunk |
| KC792054 | AGN94330 | N336 | R346 | E33 | USA | North America | Skunk |
| KC792055 | AGN94331 | N336 | R346 | E33 | USA | North America | Skunk |
| KC792062 | AGN94338 | N336 | R346 | E33 | USA | North America | Skunk |
| KC792063 | AGN94339 | N336 | R346 | E33 | USA | North America | Skunk |
| KC792064 | AGN94340 | N336 | R346 | E33 | USA | North America | Skunk |
| KC792065 | AGN94341 | N336 | R346 | E33 | USA | North America | Skunk |
| KC792066 | AGN94342 | N336 | R346 | E33 | USA | North America | Skunk |
| KC792069 | AGN94345 | N336 | R346 | E33 | USA | North America | Dog |
| KC792070 | AGN94346 | N336 | R346 | E33 | USA | North America | fox |
| KC792071 | AGN94347 | N336 | K346 | E33 | USA | North America | Eptesicus fuscus (bat) |
| KC792072 | AGN94348 | S336 | R346 | E33 | USA | North America | Cat |
| KC792074 | AGN94350 | S336 | R346 | E33 | USA | North America | Cat |
| KC792075 | AGN94351 | N336 | R346 | E33 | USA | North America | Fox |
| KC792076 | AGN94352 | D336 | R346 | E33 | USA | North America | Eptesicus fuscus |
| KC792077 | AGN94353 | N336 | K346 | E33 | USA | North America | horse |
| KC792078 | AGN94354 | N336 | R346 | E33 | USA | North America | Gray fox |
| KC792079 | AGN94355 | N336 | K346 | E33 | USA | North America | Dog |
| KC792080 | AGN94356 | N336 | K346 | E33 | USA | North America | Dog |
| KC792081 | AGN94357 | N336 | K346 | E33 | USA | North America | Cat |
| KC792082 | AGN94358 | N336 | K346 | E33 | USA | North America | Dog |
| KC792086 | AGN94362 | N336 | R346 | E33 | USA | North America | Skunk |
| KC792087 | AGN94363 | N336 | R346 | E33 | USA | North America | Skunk |
| KC792088 | AGN94364 | N336 | R346 | E33 | USA | North America | Skunk |
| KC792089 | AGN94365 | N336 | R346 | E33 | USA | North America | Skunk |
| KC792090 | AGN94366 | N336 | R346 | E33 | USA | North America | Skunk |
| KC792098 | AGN94374 | S336 | R346 | E33 | USA | North America | Goat |
| KC792099 | AGN94375 | N336 | S346 | E33 | USA | North America | Homo sapiens |
| KC792100 | AGN94376 | N336 | R346 | E33 | USA | North America | Cat |
| KC792101 | AGN94377 | D336 | R346 | E33 | USA | North America | Eptesicus fuscus |
| KC792102 | AGN94378 | S336 | R346 | E33 | USA | North America | Coyote |
| KC792103 | AGN94379 | S336 | R346 | E33 | USA | North America | Cat |
| kc792104 | AGN94380 | N336 | R346 | E33 | USA | North America | Dog |
| KC792105 | AGN94381 | N336 | K346 | E33 | USA | North America | Myotis sp |
| KC792106 | AGN94382 | D336 | R346 | E33 | USA | North America | Eptesicus fuscus |
| KC792108 | AGN94384 | D336 | R346 | E33 | USA | North America | Eptesicus fuscus |
| KC792109 | AGN94385 | D336 | R346 | E33 | USA | North America | Eptesicus fuscus |
| KC792110 | AGN94386 | N336 | E346 | D33 | USA | North America | Lasiurus borealis |
| KC792111 | AGN94387 | N336 | K346 | E33 | USA | North America | Tadarida brasiliensis |
| KC792112 | AGN94388 | N336 | E346 | D33 | USA | North America | Lasiurus borealis |
| KC792113 | AGN94389 | D336 | R346 | E33 | USA | North America | Eptesicus fuscus |
| KC792114 | AGN94390 | N336 | R346 | E33 | USA | North America | Skunk |
| KC792115 | AGN94391 | N336 | R346 | E33 | USA | North America | Skunk |
| KC792116 | AGN94392 | N336 | R346 | E33 | USA | North America | Skunk |
| KC792117 | AGN94393 | N336 | R346 | E33 | USA | North America | Skunk |
| KC792118 | AGN94394 | N336 | R346 | E33 | USA | North America | Skunk |
| KC792119 | AGN94395 | N336 | R346 | E33 | USA | North America | Skunk |
| KC792120 | AGN94396 | N336 | R346 | E33 | USA | North America | Skunk |
| KC792121 | AGN94397 | N336 | R346 | E33 | USA | North America | Skunk |
| KC792122 | AGN94398 | D336 | R346 | E33 | USA | North America | Eptesicus fuscus |
| KC792123 | AGN94399 | N336 | S346 | E33 | USA | North America | Bat |
| KC792124 | AGN94400 | N336 | K346 | E33 | USA | North America | Tadarida brasiliensis |
| KC792125 | AGN94401 | D336 | R346 | E33 | USA | North America | Eptesicus fuscus |
| KC792126 | AGN94402 | N336 | E346 | D33 | USA | North America | Lasiurus borealis |
| KC792127 | AGN94403 | D336 | R346 | E33 | USA | North America | Eptesicus fuscus |
| KC792129 | AGN94405 | N336 | K346 | E33 | USA | North America | Eptesicus fuscus |
| KC792130 | AGN94406 | N336 | S346 | E33 | USA | North America | Parastrellus hesperus |
| KC792131 | AGN94407 | N336 | S346 | E33 | USA | North America | Parastrellus hesperus |
| KC792132 | AGN94408 | N336 | K346 | E33 | USA | North America | Eptesicus fuscus |
| KC792133 | AGN94409 | N336 | K346 | E33 | USA | North America | Eptesicus fuscus |
| KC792134 | AGN94410 | N336 | E346 | D33 | USA | North America | Lasiurus borealis |
| KC792135 | AGN94411 | D336 | R346 | E33 | USA | North America | Eptesicus fuscus |
| KC792136 | AGN94412 | N336 | R346 | E33 | USA | North America | Skunk |
| KC792138 | AGN94414 | N336 | R346 | E33 | USA | North America | Skunk |
| KC792139 | AGN94415 | N336 | R346 | E33 | USA | North America | Skunk |
| KC792140 | AGN94416 | N336 | R346 | E33 | USA | North America | Skunk |
| KC792141 | AGN94417 | N336 | R346 | E33 | USA | North America | Skunk |
| KC792142 | AGN94418 | N336 | R346 | E33 | USA | North America | Skunk |
| KC792144 | AGN94420 | N336 | K346 | E33 | USA | North America | Eptesicus fuscus |
| KC792145 | AGN94421 | N336 | K346 | E33 | USA | North America | Bat |
| KC792146 | AGN94422 | N336 | R346 | E33 | USA | North America | Skunk |
| KC792148 | AGN94424 | N336 | R346 | E33 | USA | North America | Red fox |
| KC792149 | AGN94425 | N336 | S346 | E33 | USA | North America | Parastrellus hesperus |
| KC792150 | AGN94426 | N336 | S346 | E33 | USA | North America | Parastrellus hesperus |
| KC792151 | AGN94427 | N336 | S346 | E33 | USA | North America | Parastrellus hesperus |
| KC792152 | AGN94428 | N336 | R346 | E33 | USA | North America | Dog |
| KC792153 | AGN94429 | N336 | R346 | E33 | USA | North America | Red fox |
| KC792154 | AGN94430 | N336 | R346 | E33 | USA | North America | Red fox |
| KC792155 | AGN94431 | N336 | R346 | E33 | USA | North America | Red fox |
| KC792156 | AGN94432 | N336 | R346 | E33 | USA | North America | Arctic fox |
| KC792157 | AGN94433 | N336 | R346 | E33 | USA | North America | Red fox |
| KC792158 | AGN94434 | N336 | R346 | E33 | USA | North America | Red fox |
| KC792159 | AGN94435 | N336 | R346 | E33 | USA | North America | Red fox |
| KC792160 | AGN94436 | N336 | R346 | E33 | USA | North America | Arctic fox |
| KC792161 | AGN94437 | N336 | R346 | E33 | USA | North America | Dog |
| KC792162 | AGN94438 | N336 | R346 | E33 | USA | North America | Red fox |
| KC792163 | AGN94439 | N336 | R346 | E33 | USA | North America | Red fox |
| KC792164 | AGN94440 | N336 | R346 | E33 | USA | North America | Red fox |
| KC792165 | AGN94441 | N336 | R346 | E33 | USA | North America | Arctic fox |
| KC792166 | AGN94442 | N336 | R346 | E33 | USA | North America | Wolf |
| KC792167 | AGN94443 | N336 | R346 | E33 | USA | North America | Arctic fox |
| KC792168 | AGN94444 | N336 | R346 | E33 | USA | North America | Red fox |
| KC792169 | AGN94445 | N336 | R346 | E33 | USA | North America | Red fox |
| KC792170 | AGN94446 | N336 | R346 | E33 | USA | North America | Dog |
| KC792171 | AGN94447 | N336 | R346 | E33 | USA | North America | Red fox |
| KC792172 | AGN94448 | N336 | R346 | E33 | USA | North America | Red fox |
| KC792173 | AGN94449 | N336 | R346 | E33 | USA | North America | Red fox |
| KC792174 | AGN94450 | N336 | R346 | E33 | USA | North America | Arctic fox |
| KC792175 | AGN94451 | N336 | R346 | E33 | USA | North America | Arctic fox |
| KC792176 | AGN94452 | N336 | R346 | E33 | USA | North America | Red fox |
| KC792177 | AGN94453 | N336 | R346 | E33 | USA | North America | Arctic fox |
| KC792178 | AGN94454 | N336 | R346 | E33 | USA | North America | Arctic fox |
| KC792179 | AGN94455 | N336 | R346 | E33 | USA | North America | Arctic fox |
| KC792180 | AGN94456 | N336 | R346 | E33 | USA | North America | Arctic fox |
| KC792181 | AGN94457 | N336 | R346 | E33 | USA | North America | Arctic fox |
| KC792182 | AGN94458 | N336 | R346 | E33 | USA | North America | Red fox |
| KC792183 | AGN94459 | N336 | R346 | E33 | USA | North America | Arctic fox |
| KC792184 | AGN94460 | N336 | R346 | E33 | USA | North America | Red fox |
| KC792185 | AGN94461 | N336 | R346 | E33 | USA | North America | Arctic fox |
| KC792186 | AGN94462 | N336 | R346 | E33 | USA | North America | Red fox |
| KC792187 | AGN94463 | N336 | R346 | E33 | USA | North America | Red fox |
| KC792188 | AGN94464 | S336 | R346 | E33 | USA | North America | Arctic fox |
| KC792189 | AGN94465 | N336 | K346 | E33 | USA | North America | Tadarida brasiliensis |
| KC792190 | AGN94466 | N336 | K346 | E33 | USA | North America | Tadarida brasiliensis |
| KC792191 | AGN94467 | N336 | R346 | E33 | USA | North America | Skunk |
| KC792192 | AGN94468 | N336 | R346 | E33 | USA | North America | Skunk |
| KC792193 | AGN94469 | N336 | R346 | E33 | USA | North America | Skunk |
| KC792194 | AGN94470 | N336 | R346 | E33 | USA | North America | Skunk |
| KC792195 | AGN94471 | N336 | R346 | E33 | USA | North America | Skunk |
| KC792196 | AGN94472 | N336 | R346 | E33 | Mexico | North America | n.a |
| KC792197 | AGN94473 | N336 | R346 | E33 | USA | North America | Skunk |
| KC792201 | AGN94477 | N336 | R346 | E33 | Mexico | North America | Homo sapiens |
| KC792205 | KC792205 | N336 | R346 | E33 | USA | North America | Skunk |
| KC792209 | AGN94485 | N336 | R346 | E33 | USA | North America | Skunk |
| KC792210 | AGN94486 | N336 | R346 | E33 | USA | North America | Skunk |
| KC792211 | AGN94487 | N336 | R346 | E33 | USA | North America | Skunk |
| KC792212 | AGN94488 | N336 | R346 | E33 | USA | North America | Skunk |
| KC792213 | AGN94489 | N336 | R346 | E33 | USA | North America | Skunk |
| KC792214 | AGN94490 | N336 | E346 | D33 | USA | North America | Lasiurus cinereus |
| KC792215 | AGN94491 | N336 | R346 | E33 | USA | North America | Dog |
| KC792216 | AGN94492 | N336 | R346 | E33 | USA | North America | Skunk |
| KC792217 | AGN94493 | N336 | R346 | E33 | USA | North America | Cat |
| KC792218 | AGN94494 | N336 | R346 | E33 | USA | North America | Skunk |
| KC792219 | AGN94495 | N336 | R346 | E33 | USA | North America | mongoose |
| KC792237 | AGN94513 | N336 | R346 | E33 | USA | North America | n.a |
| KC792238 | AGN94514 | N336 | R346 | E33 | USA | North America | Homo sapiens |
| KC792239 | AGN94515 | N336 | R346 | E33 | USA | North America | Cat |
| KC792241 | AGN94517 | N336 | E346 | D33 | USA | North America | Lasiurus borealis |
| KC792258 | AGN94534 | N336 | R346 | E33 | Mexico | North America | Dog |
| KC792259 | AGN94535 | N336 | R346 | E33 | Mexico | North America | Desmodus rotundus |
| KC792261 | AGN94537 | N336 | E346 | D33 | USA | North America | Lasiurus borealis |
| KC792262 | AGN94538 | N336 | E346 | D33 | USA | North America | Lasiurus borealis |
| KC792263 | AGN94539 | N336 | E346 | D33 | USA | North America | Lasiurus borealis |
| KC792264 | AGN94540 | N336 | E346 | D33 | USA | North America | Lasiurus cinereus |
| KC792266 | AGN94542 | N336 | R346 | E33 | USA | North America | hognosed skunk |
| KC792267 | AGN94543 | N336 | R346 | E33 | USA | North America | Skunk |
| KC792268 | AGN94544 | N336 | R346 | E33 | USA | North America | Skunk |
| KC792269 | AGN94545 | N336 | R346 | E33 | USA | North America | Spotted skunk |
| KC792270 | AGN94546 | N336 | R346 | E33 | USA | North America | Skunk |
| KC792271 | AGN94547 | N336 | R346 | E33 | USA | North America | Skunk |
| KC792272 | AGN94548 | N336 | R346 | E33 | USA | North America | Puma |
| KC792273 | AGN94549 | N336 | R346 | E33 | USA | North America | Skunk |
| KC792274 | AGN94550 | N336 | R346 | E33 | USA | North America | Skunk |
| KC792275 | AGN94551 | N336 | E346 | D33 | USA | North America | Lasiurus borealis |
| KC792276 | AGN94552 | N336 | E346 | D33 | USA | North America | Lasiurus borealis |
| KF484518 | AHB86362 | N336 | R346 | E33 | USA | North America | Skunk |
| KF484519 | AHB86363 | N336 | R346 | E33 | USA | North America | Skunk |
| KF484520 | AHB86364 | N336 | R346 | E33 | USA | North America | Skunk |
| KF484521 | AHB86365 | N336 | R346 | E33 | USA | North America | Skunk |
| KF484522 | AHB86366 | N336 | R346 | E33 | USA | North America | Skunk |
| KF484523 | AHB86367 | N336 | R346 | E33 | USA | North America | Skunk |
| KF484524 | AHB86368 | N336 | R346 | E33 | USA | North America | Skunk |
| KF484525 | AHB86369 | N336 | R346 | E33 | USA | North America | Mephitis macroura |
| KF484526 | AHB86370 | N336 | R346 | E33 | USA | North America | Procyon lotor |
| KF484527 | AHB86371 | N336 | R346 | E33 | USA | North America | Skunk |
| KF484528 | AHB86372 | N336 | R346 | E33 | USA | North America | Skunk |
| KF484529 | AHB86373 | N336 | R346 | E33 | USA | North America | Skunk |
| KF484530 | AHB86374 | N336 | R346 | E33 | USA | North America | Skunk |
| KF484531 | AHB86375 | N336 | R346 | E33 | USA | North America | Skunk |
| KF484532 | AHB86376 | N336 | R346 | E33 | USA | North America | Skunk |
| KF484533 | AHB86377 | N336 | R346 | E33 | USA | North America | Skunk |
| KF484534 | AHB86378 | N336 | R346 | E33 | USA | North America | Skunk |
| KF484535 | AHB86379 | N336 | R346 | E33 | USA | North America | Skunk |
| KF484536 | AHB86380 | N336 | R346 | E33 | USA | North America | Skunk |
| KF484537 | AHB86381 | N336 | R346 | E33 | USA | North America | Skunk |
| KF484538 | AHB86382 | N336 | R346 | E33 | USA | North America | Skunk |
| KF484539 | AHB86383 | N336 | R346 | E33 | USA | North America | Canis familiaris |
| KF484540 | AHB86384 | N336 | R346 | E33 | USA | North America | Skunk |
| KF484541 | AHB86385 | N336 | R346 | E33 | USA | North America | Skunk |
| KF484542 | AHB86386 | N336 | R346 | E33 | USA | North America | Bos taurus |
| KF484543 | AHB86387 | S336 | R346 | E33 | USA | North America | Skunk |
| KF484544 | AHB86388 | N336 | R346 | E33 | USA | North America | Skunk |
| KF484545 | AHB86389 | N336 | R346 | E33 | USA | North America | Skunk |
| KF484546 | AHB86390 | N336 | R346 | E33 | USA | North America | Felis catus |
| KF484547 | AHB86391 | N336 | R346 | E33 | USA | North America | Felis catus |
| KF484548 | AHB86392 | N336 | R346 | E33 | USA | North America | Felis catus |
| KF484549 | AHB86393 | N336 | R346 | E33 | USA | North America | Skunk |
| KF484550 | AHB86394 | N336 | R346 | E33 | USA | North America | Skunk |
| KF484551 | AHB86395 | N336 | R346 | E33 | USA | North America | Skunk |
| KF484552 | AHB86396 | N336 | R346 | E33 | USA | North America | Skunk |
| KF484553 | AHB86397 | N336 | R346 | E33 | USA | North America | Skunk |
| KF484554 | AHB86398 | N336 | R346 | E33 | USA | North America | Skunk |
| KF484555 | AHB86399 | N336 | R346 | E33 | USA | North America | Skunk |
| KF484556 | AHB86400 | N336 | R346 | E33 | USA | North America | Skunk |
| KF484557 | AHB86401 | N336 | R346 | E33 | USA | North America | Skunk |
| KF484558 | AHB86402 | N336 | R346 | E33 | USA | North America | Canis familiaris |
| KF484559 | AHB86403 | N336 | R346 | E33 | USA | North America | Skunk |
| KF484566 | AHB86410 | N336 | R346 | E33 | USA | North America | Skunk |
| KJ174608 | AIL00980 | N336 | K346 | E33 | USA | North America | Eptesicus fuscus |
| KJ174609 | AIL00981 | N336 | K346 | E33 | USA | North America | Eptesicus fuscus |
| KJ174610 | AIL00982 | N336 | K346 | E33 | USA | North America | Eptesicus fuscus |
| KJ174611 | AIL00983 | N336 | K346 | E33 | USA | North America | Eptesicus fuscus |
| KJ174612 | AIL00984 | N336 | K346 | E33 | USA | North America | Eptesicus fuscus |
| KJ174613 | AIL00985 | N336 | K346 | E33 | USA | North America | Eptesicus fuscus |
| KJ174614 | AIL00986 | N336 | K346 | E33 | USA | North America | Eptesicus fuscus |
| KJ174615 | AIL00987 | N336 | K346 | E33 | USA | North America | Eptesicus fuscus |
| KJ174616 | AIL00988 | N336 | K346 | E33 | USA | North America | Eptesicus fuscus |
| KJ174617 | AIL00989 | N336 | K346 | E33 | USA | North America | Eptesicus fuscus |
| KJ174618 | AIL00990 | D336 | R346 | E33 | USA | North America | Eptesicus fuscus |
| KJ174619 | AIL00991 | D336 | R346 | E33 | USA | North America | Eptesicus fuscus |
| KJ174620 | AIL00992 | D336 | R346 | E33 | USA | North America | Eptesicus fuscus |
| KJ174621 | AIL00993 | D336 | R346 | E33 | USA | North America | Eptesicus fuscus |
| KJ174622 | AIL00994 | D336 | R346 | E33 | USA | North America | Eptesicus fuscus |
| KJ174623 | AIL00995 | D336 | R346 | E33 | USA | North America | Lasiurus borealis |
| KJ174624 | AIL00996 | D336 | R346 | E33 | USA | North America | Eptesicus fuscus |
| KJ174625 | AIL00997 | D336 | R346 | E33 | USA | North America | Eptesicus fuscus |
| KJ174626 | AIL00998 | D336 | R346 | E33 | USA | North America | Eptesicus fuscus |
| KJ174627 | AIL00999 | N336 | E346 | D33 | USA | North America | Lasiurus borealis |
| KJ174628 | AIL01000 | N336 | E346 | D33 | USA | North America | Lasiurus borealis |
| KJ174629 | AIL01001 | N336 | E346 | D33 | USA | North America | Lasiurus borealis |
| KJ174630 | AIL01002 | N336 | E346 | D33 | USA | North America | Lasiurus borealis |
| KJ174631 | AIL01003 | N336 | E346 | D33 | USA | North America | Lasiurus borealis |
| KJ174632 | AIL01004 | N336 | E346 | D33 | USA | North America | Lasiurus borealis |
| KJ174633 | AIL01005 | N336 | E346 | D33 | USA | North America | Tadarida brasiliensis |
| KJ174634 | AIL01006 | N336 | E346 | D33 | USA | North America | Lasiurus borealis |
| KJ174635 | AIL01007 | N336 | E346 | D33 | USA | North America | Lasiurus borealis |
| KJ174636 | AIL01008 | N336 | E346 | D33 | USA | North America | Perimyotis subflavus |
| KJ174637 | AIL01009 | N336 | E346 | D33 | USA | North America | Lasiurus borealis |
| KJ174638 | AIL01010 | N336 | E346 | D33 | USA | North America | Lasiurus borealis |
| KJ174639 | AIL01011 | N336 | E346 | D33 | USA | North America | Lasiurus borealis |
| KJ174640 | AIL01012 | N336 | E346 | D33 | USA | North America | Lasiurus borealis |
| KJ174641 | AIL01013 | N336 | E346 | D33 | USA | North America | Myotis velifer |
| KJ174642 | AIL01014 | N336 | E346 | D33 | USA | North America | Lasiurus borealis |
| KJ174643 | AIL01015 | N336 | E346 | D33 | USA | North America | Lasiurus cinereus |
| KJ174644 | AIL01016 | N336 | E346 | D33 | USA | North America | Lasiurus cinereus |
| KJ174645 | AIL01017 | N336 | K346 | E33 | USA | North America | Lasiurus intermedius |
| KJ174646 | AIL01018 | N336 | K346 | E33 | USA | North America | Lasionycteris noctivagans |
| KJ174647 | AIL01019 | N336 | K346 | E33 | USA | North America | Myotis californicus |
| KJ174648 | AIL01020 | N336 | E346 | D33 | USA | North America | Lasiurus xanthinus |
| KJ174649 | AIL01021 | N336 | E346 | D33 | USA | North America | Lasiurus ega |
| KJ174650 | AIL01022 | N336 | K346 | E33 | USA | North America | Myotis velifer |
| KJ174651 | AIL01023 | N336 | K346 | E33 | USA | North America | Myotis velifer |
| KJ174652 | AIL01024 | N336 | K346 | E33 | USA | North America | Myotis californicus |
| KJ174653 | AIL01025 | N336 | S346 | E33 | USA | North America | Myotis californicus |
| KJ174654 | AIL01026 | N336 | S346 | E33 | USA | North America | Urocyon cinereoargenteus |
| KJ174655 | AIL01027 | N336 | S346 | E33 | USA | North America | Urocyon cinereoargenteus |
| KJ174656 | AIL01028 | N336 | K346 | E33 | USA | North America | Vulpes vulpes |
| KJ174657 | AIL01029 | N336 | K346 | E33 | USA | North America | Vulpes vulpes |
| KJ174658 | AIL01030 | N336 | S346 | E33 | USA | North America | Myotis lucifugus |
| KJ174659 | AIL01031 | N336 | E346 | D33 | USA | North America | Vulpes vulpes |
| KJ174660 | AIL01032 | D336 | R346 | E33 | USA | North America | Nycticeius humeralis |
| KJ174661 | AIL01033 | N336 | K346 | E33 | USA | North America | Canis familiaris |
| KJ174662 | AIL01034 | N336 | K346 | E33 | USA | North America | Felis catus |
| KJ174663 | AIL01035 | N336 | S346 | E33 | USA | North America | Parastrellus hesperus |
| KJ174664 | AIL01036 | N336 | S346 | E33 | USA | North America | Parastrellus hesperus |
| KJ174665 | AIL01037 | N336 | S346 | E33 | USA | North America | Parastrellus hesperus |
| KJ174666 | AIL01038 | N336 | K346 | E33 | USA | North America | Perimyotis subflavus |
| KJ174667 | AIL01039 | N336 | K346 | E33 | USA | North America | Tadarida brasiliensis |
| KJ174668 | AIL01040 | N336 | K346 | E33 | USA | North America | Tadarida brasiliensis |
| KJ174669 | AIL01041 | N336 | K346 | E33 | USA | North America | Tadarida brasiliensis |
| KJ174670 | AIL01042 | N336 | K346 | E33 | USA | North America | Tadarida brasiliensis |
| KJ174671 | AIL01043 | N336 | K346 | E33 | USA | North America | Myotis lucifugus |
| KJ174672 | AIL01044 | N336 | K346 | E33 | USA | North America | Eptesicus fuscus |
| KJ174673 | AIL01045 | N336 | K346 | E33 | USA | North America | Tadarida brasiliensis |
| KJ174674 | AIL01046 | N336 | K346 | E33 | USA | North America | Tadarida brasiliensis |
| KJ174675 | AIL01047 | N336 | K346 | E33 | USA | North America | Eptesicus fuscus |
| KJ174676 | AIL01048 | N336 | K346 | E33 | USA | North America | Eptesicus fuscus |
| KJ174677 | AIL01049 | N336 | K346 | E33 | USA | North America | Eptesicus fuscus |
| KJ174678 | AIL01050 | N336 | K346 | E33 | USA | North America | Eptesicus fuscus |
| KJ174679 | AIL01051 | N336 | K346 | E33 | USA | North America | Eptesicus fuscus |
| KJ174680 | AIL01052 | N336 | K346 | E33 | USA | North America | Eptesicus fuscus |
| KJ174681 | AIL01053 | N336 | K346 | E33 | USA | North America | Urocyon cinereoargenteus |
| KJ174682 | AIL01054 | N336 | K346 | E33 | USA | North America | Myotis lucifugu |
| KJ174683 | AIL01055 | N336 | K346 | E33 | USA | North America | Canis latrans |
| RVU11737 | AAA64544 | N336 | R346 | E33 | Canada | North America | Arctic fox |
| U11736 | AAA64543 | N336 | R346 | E33 | Canada | North America | Arctic fox |
| U11739 | AAA64546 | N336 | R346 | E33 | Canada | North America | Vulpes vulpes |
| U11741 | AAA64548 | N336 | R346 | E33 | Canada | North America | Arctic fox |
| U11742 | AAA64549 | N336 | R346 | E33 | Canada | North America | Vulpes vulpes |
| U11743 | AAA64550 | N336 | R346 | E33 | Canada | North America | Arctic fox |
| U11744 | AAA64551 | N336 | R346 | E33 | Canada | North America | Arctic fox |
| U11745 | AAA64552 | N336 | R346 | E33 | Canada | North America | Arctic fox |
| U11746 | AAA64553 | N336 | R346 | E33 | Canada | North America | Vulpes vulpes |
| U11747 | AAA64554 | N336 | R346 | E33 | Canada | North America | Vulpes vulpes |
| U11748 | AAA64555 | N336 | R346 | E33 | Canada | North America | Vulpes vulpes |
| U11750 | AAA64557 | N336 | R346 | E33 | Canada | North America | Vulpes vulpes |
| U11751 | AAA64558 | N336 | R346 | E33 | Canada | North America | Arctic fox |
| U11752 | AAA64559 | N336 | R346 | E33 | Canada | North America | Vulpes vulpes |
| U52946 | AAA97953 | N336 | K346 | E33 | USA | North America | Silver-haired bat |
| KC791792 | AGN94068 | N336 | K346 | N/A | USA | North America | spotted skunk |
| KC791802 | AGN94078 | N336 | K346 | N/A | USA | North America | Eptesicus fuscus |
| KC792128 | AGN94404 | N336 | K346 | N/A | USA | North America | Tadarida brasiliensis |
| KC792137 | AGN94413 | N336 | R346 | N/A | USA | North America | Skunk |
| KC792202 | AGN94478 | N336 | K346 | N/A | Mexico | North America | Homo sapiens |
| KC792277 | AGN94553 | N336 | K346 | N/A | USA | North America | Skunk |
| KC792083 | AGN94359 | N/A | N/A | E33 | USA | North America | Skunk |
| KC792084 | AGN94360 | N/A | N/A | E33 | USA | North America | Skunk |
| KC792085 | AGN94361 | N/A | N/A | E33 | USA | North America | Skunk |
| KC792107 | AGN94383 | N/A | N/A | E33 | USA | North America | Eptesicus fuscus |
| KC792221 | AGN94497 | N/A | N/A | E33 | Mexico | North America | Skunk |
